# Standardised self-management kits for children with type 1 diabetes: pragmatic randomised trial of effectiveness and cost-effectiveness

**DOI:** 10.1136/bmjopen-2019-032163

**Published:** 2020-03-12

**Authors:** Jane Noyes, Davina Allen, Cynthia Carter, Deborah Edwards, Rhiannon Tudor Edwards, Daphne Russell, Ian T Russell, Llinos Haf Spencer, Yvonne Sylvestre, Rhiannon Whitaker, Seow Tien Yeo, John W Gregory

**Affiliations:** 1 School of Health Sciences, Bangor University, Bangor, UK; 2 School of Healthcare Sciences, Cardiff University, Cardiff, UK; 3 School of Journalism, Media and Culture, Cardiff University, Cardiff, UK; 4 Centre for Health Economics and Medicines Management Evaluation, School of Health Sciences, Bangor University, Bangor, UK; 5 Swansea University Medical School, Swansea University, Swansea, UK; 6 Manchester Academic Health Science (MAHSC) Clinical Trials Unit, Christie Hospital NHS Foundation Trust, Manchester, UK; 7 Whitaker Research Limited, Rhos on Sea, North Wales, UK; 8 Division of Population Medicine, School of Medicine, Cardiff University, Cardiff, UK

**Keywords:** paediatric endocrinology, health services administration & management, health economics

## Abstract

**Objective:**

To estimate the effectiveness of standardised self-management kits for children with type 1 diabetes.

**Design:**

Pragmatic trial with randomisation ratio of two intervention: one control. Qualitative process evaluation.

**Setting:**

11 diabetes clinics in England and Wales.

**Participants:**

Between February 2010 and August 2011, we validly randomised 308 children aged 6–18 years; 201 received the intervention.

**Intervention:**

We designed kits to empower children to achieve glycaemic control, notably by recording blood glucose and titrating insulin. The comparator was usual treatment.

**Outcome measures at 3 and 6 months:**

Primary: Diabetes Pediatric Quality of Life Inventory (PedsQL). Secondary: HbA1c; General PedsQL; EQ-5D; healthcare resource use.

**Results:**

Of the five Diabetes PedsQL dimensions, Worry showed adjusted scores significantly favouring self-management kits at 3 months (mean child-reported difference =+5.87; Standard error[SE]=2.19; 95% confidence interval [CI]) from +1.57 to +10.18; p=0.008); but Treatment Adherence significantly favoured controls at 6 months (mean child-reported difference=−4.68; SE=1.74; 95%CI from −8.10 to −1.25; p=0.008). Intervention children reported significantly worse changes between 3 and 6 months on four of the five Diabetes PedsQL dimensions and on the total score (mean difference=−3.20; SE=1.33; 95% CI from −5.73 to −0.67; p=0.020). There was no evidence of change in HbA1c; only 18% of participants in each group achieved recommended levels at 6 months. No serious adverse reactions attributable to the intervention or its absence were reported.

Use of kits was poor. Few children or parents associated blood glucose readings with better glycaemic control. The kits, costing £185, alienated many children and parents.

**Conclusions:**

Standardised kits showed no evidence of benefit, inhibited diabetes self-management and increased worry. Future research should study relationships between children and professionals, and seek new methods of helping children and parents to manage diabetes.

**Trial registration number:**

ISRCTN17551624.

**Strengths and limitations of this study**

- The self-management kits that were tested in this trial were designed with large numbers of children and young people and their parents in a 3-year study.
- We conducted a fully powered pragmatic trial of children’s self-management kits in routine practice.
- A third of eligible children and young people declined to participate in the trial.
- Our large process evaluation provided a detailed explanation of the mechanisms that appear to lead to negative outcomes and lack of engagement by children and parents.
- The cost-effectiveness analysis was limited as there was no evidence of intervention effect.

“Kudos plain language summary”

Children and their parents find it very challenging to manage their type 1 diabetes well. Daily self-management is complex and involves adjusting the amount of insulin injected and sticking to specific foods and portion sizes to stay well. The amount of sugar in the blood should also be measured regularly to make sure that levels are not too high or too low. We worked with large numbers of children age 6-18years to design three age appropriate diabetes self-management kits. The kits contained everything that the children said that they needed to better manage their diabetes. We then tested the new self-management kits in a large trial to see if children who used the kits were better able to manage their diabetes compared to those who did not use the kits. The trial showed that the kits made no difference and in some cases their diabetes management became worse. Over one third of children who were eligible did not want to take part in the trial. The kits caused some children to worry more and alienated both children and their parents. We asked children and their parents why they did not find the kits helpful. They told us that they did not like anything that reminded them that they had diabetes. Nor did they fully understand what good diabetes management involved or the risks associated with not managing their diabetes well. Children frequently reported that they did not like attending children’s diabetes clinics or the authoritarian approach taken by diabetes professionals to their diabetes management. We concluded that there needs to be a fresh approach to the way that children’s diabetes services are organised and managed. New ideas are needed about how best to design children’s diabetes education.

## Introduction

Managing diabetes at all ages costs the National Health Service (NHS) nearly £10 billion a year; 80% of this is for managing avoidable complications.^1^ Learning to manage one’s diabetes in childhood is important to prevent long-term and potentially life-threatening complications of poor glycaemic control. Diabetes care pathways^2–5^ have been available for over a decade and the proportion of children in England and Wales who achieved the previous National Institute for Health and Care Excellence (NICE) target of an HbA1c level of ≤58 mmol/mol has slowly increased from 14.5% in 2009 to 15.8% in 2013 and 26.6% in 2015.^6–8^ In 2015, NICE further amended the target to ≤48 mmol/mol.^9^ Schools vary considerably in the support given to children to manage their insulin administration, diet and participation in extracurricular activities and sports.^10^Achieving optimal glycaemic control is most difficult during transition from paediatric to adult services when young people become independent of parents and families.^11^

Optimal diabetes self-management requires titration of insulin doses against blood glucose levels, dietary intake and planned physical activities.^3 9^ This skill is essential for children to participate fully in school life and social activities outside school.^10^Models of children’s diabetes care emphasise a family-centred approach with intensive education and support following diagnosis, with increasing responsibility for care transferred to the child over time.^6 7^ There has been no standardisation of diabetes self-management information given to children to use at home and school.

To prepare for the trial, research was undertaken in the current and a previous study with children, young people and parents to identify the types and formats of self-management information likely to inspire behaviour change in children and young people with diabetes.^12 13^ Our systematic review of educational and support interventions to improve diabetes self-management in schools revealed no effective interventions, but many barriers to self-management when children were away from their parents.^10^ Hence, the goal of this trial was to evaluate whether standardised age-appropriate self-management kits motivate children and their families to avoid complications caused by uncontrolled blood sugar levels. Our primary aim was to assess whether the kits enabled children to manage their type 1 diabetes by titrating their insulin dose against regular blood glucose readings. Our secondary aim was to assess how children, their families and diabetes professionals perceived and used these kits.

The subsequent availability of published reports for five other contemporaneous trials of UK children’s diabetes education interventions created a new opportunity to review all six trials and explore why none of these six interventions had any effect. In particular, since the original report was published in the NIHR journals library,^12^ we have now reanalysed HbA1c, a secondary outcome measure in our trial so that results can be discussed in relation to five contemporaneous UK trials and other international studies. We have also undertaken a more detailed analysis of the subdomains of Diabetes Pediatric Quality of Life Inventory (PedsQL), the primary outcome measure of our trial, to better understand the benefits and harms of the intervention.

## Methods

### Study design and participants

We conducted a pragmatic randomised trial, including economic and process evaluations, in NHS paediatric diabetes clinics in England and Wales.^12–14^ The Medicines for Children Research Network (England) and the Children and Young People Research Network (Wales) recruited diabetes multidisciplinary teams in 11 NHS District General Hospitals.

### Intervention

Children in the comparator group received treatment as usually provided in each of the 11 diabetes clinics.^14^ A record of ‘usual care’ was made in order to have a clear idea of the comparator with which EPIC was being compared. Children in the intervention group received a standardised but flexible self-management kit known as ‘Evidence into Practice—Information Counts’ (EPIC). Following extensive literature review, consultation and fieldwork,^10 12–14^ EPIC kits comprised the following.

- Three age-specific (6–10 years, 11–15 or 16–18) diabetes self-management kits comprising booklets, magazines, leaflets, CDs and website links.
- Three corresponding diaries for those using insulin injections.^*^
- One diary for children using insulin pumps.^*^
- Sheets for recording carbohydrate intake.
- Stickers (6–15 years) and marker pens (11–15 years) for children to personalise their folder.

*Children could also use blood glucose recordings downloaded from their blood glucose monitors.

A detailed account of intervention development and the theoretical basis of the EPIC intervention is reported elsewhere.^12^ In brief, we designed EPIC kits so that children and young people had relevant information to self-manage their diabetes (with support from parents for younger children) consistent with relevant clinical guidelines,^2–5^ incorporating age-appropriate preferences for information^13^ and consistent with clinical practice in the UK NHS. Key features intended to appeal to children and engage them in EPIC kits included presentation to and ownership by the child; age-appropriate messages stressing ‘top 10 tips’ for self-management; the invitation ‘take me with you wherever you go’; contextual questions about self-management and life-style; integration into routine encounters with the child’s multidisciplinary team, especially the Paediatrician and the Paediatric Diabetes Specialist Nurse (PDSN); scope for professionals and parents to tailor EPIC kits to each child and review at subsequent appointments; and encouraging the child to record blood glucose and insulin titration every day to share with professionals. We invited diabetes team members to attend training in their hospital which described EPIC kits, introduced the manual and suggested how to engage each child, and how to integrate EPIC kits into routine care.

### Random allocation

Between February 2010 and August 2011, we screened diabetes outpatient clinic lists in 11 hospitals for potentially eligible children between 6 and 18 years with type 1 diabetes. We excluded children with communication difficulties, needle phobia or other impairments judged inconsistent with the trial. We sent invitation letters and age-specific information sheets to families of eligible children. Research nurses independent of both clinical and research teams sought written informed consent to the trial from parents and children over 16 years, or assent from children under 16 years. Consenting parents and their children provided baseline data.

Research nurses then used a secure web-based dynamic randomisation system^15^ to allocate children at random between EPIC and treatment as usual, stratified by hospital, age, gender and whether 2 years had elapsed since diagnosis; the allocation ratio was two intervention participants for every control. These nurses told children’s clinical teams of these allocations so they could initiate EPIC at the next consultation. We followed these children for 6 months.

### Masking

Though it was neither desirable nor feasible to blind clinical staff or participants to treatment allocated, we sought to blind assessors. Analysis was undertaken by an independent trial support unit.

### Outcome measures

The primary outcome was children’s self-efficacy in coping with their diabetes, measured by child and (proxy) parent versions of the Diabetes PedsQL^16^ 6 months after randomisation, with interim scores at 3 months. The Diabetes PedsQL comprises 33 items (32 for younger children) covering five domains—diabetes, treatment adherence, treatment barriers, communication and worry. The resulting scores lie between 0 and 100 with higher scores indicating better coping.

Secondary outcomes comprised: HbA1c measured at routine quarterly clinics; health-related quality of life measured by child and (proxy) parent versions of the General PedsQL^17^ and health-related quality of life measured by the EuroQol 5 Dimension 3 level (EQ-5D-3L).^18^ We used the youth version for children under 16 years, the adult version for those over 16 and parents’ proxy scores for all children. Follow-up questionnaires, completed after 3 and 6 months in clinic or by post, also sought data on episodes of diabetic ketoacidosis and health service use, especially hospital admissions for acute complications, recorded on a diabetes-specific version of the Client Service Receipt Inventory.^19^ We checked health service use against children’s hospital notes.

Children and parents also completed baseline questionnaires covering sociodemographic characteristics and the duration and self-management of their diabetes. Children received £10 vouchers for each questionnaire they completed at 3 or 6 months. Non-responders received both telephone and postal reminders after 2 and 4 weeks.

We defined serious adverse events (SAEs) as adverse events that, in the judgement of the relevant site Principal Investigator, were lethal, life threatening, resulting in hospital admission, resulting in persistent or significant disability or incapacity, or otherwise medically significant. We defined serious adverse reactions (SARs) as SAEs that, in the judgement of the Clinical Principal Investigator (CPI) and research team, were definitely, probably or possibly related to the EPIC intervention or to treatment as usual.

### Sample size

To yield 80% power of detecting an effect size of 0.4 in the primary outcome of self-efficacy when using a two-sided 5% significance level, we aimed to analyse 202 children, initially by recruiting 252 children—168 allocated to EPIC and 84 controls, thus allowing for losing 20% of participants to follow-up.^20 21^ As fewer participants than expected initially responded to questionnaires, we reviewed these calculations in consultation with the Data Monitoring Committee and increased the target to 337 to allow for losing 40% to follow-up. We also introduced monetary vouchers for completed questionnaires.

### Statistical analyses

Analysis was by treatment allocated. We imputed missing quality of life data in accordance with published guidance for each measure.^22 23^ We used the fully conditional specification technique and five multiple imputations across time points to impute these data.^22^ We compared differences between treatment groups using mixed models to undertake repeated-measures analysis of variance, adjusting for stratification variables and baseline values. We estimated parameters for three fixed factors—the time-points of 3 and 6 months and treatment group. We modelled hospital as a random factor. We included the interaction between treatment group and time-point to test whether differences between treatment groups varied between time-points. These analyses modelled diabetes self-efficacy (Diabetes PedsQL), quality of life (General PedsQL) and health utility (EQ-5D), both to study change in individuals, and in cohort analysis to compare change in group means.

### Economic analysis

We costed the age-specific EPIC kits by recording quantities and costs of materials used to produce them, and estimating the mean additional time taken by PDSNs. We collected retrospective data on children’s use of primary and secondary healthcare services over the previous 3 months. We applied national unit costs in 2010–2011 pounds sterling (£) to these services.^24 25^ As we followed participants for only 6 months, we did not discount costs or effects.^26^ We undertook cost-consequence analysis from an NHS perspective and tested the sensitivity of findings to the substitution of consultants for nurses in presenting the EPIC kits.

### Process evaluation

After the trial, we recruited a second sample for semistructured recorded interviews in depth. The process evaluation was conducted up to December 2013. We used purposive sampling to generate maximum variation in the ages, genders, times since diagnosis and types of insulin transfer (injection or pump) of 41 children allocated to EPIC, and 19 comparator children. We also interviewed 66 parents and family members of these children. These interviews explored: views and experiences of both EPIC kits and treatment as usual; how participants managed self-care at home and in school and other social contexts; and children’s interactions with diabetes teams. Before the trial, we interviewed professionals in each of the 11 hospitals about their previous practice; after the trial, we surveyed them by post about how they had implemented EPIC. We recorded interviews, transcribed them verbatim and analysed them using the thematic framework approach.^27^ We mapped the resulting themes onto the underlying theory and developed higher level themes and understanding in depth.

As part of the process evaluation, we also undertook a discourse analysis of a purposive sample of children’s diabetes resources used in the UK NHS.^28^ Discourse analysis is a way of identifying and analysing the assumptions made by information sources about their relationships with their readership, assumptions that had shaped the messages of the selected resources, some of which had appeared in the intervention pack and may also have been available to comparator children.

### Patient and public involvement

Children, parents and public representatives were actively involved in an extensive 3-year prior study, as well as the current study to develop the educational intervention.^12^ A core set of diabetes education materials were codesigned by children with type 1 diabetes of various ages. Children and parents were represented in the trial advisory group. All participants received a child-centred copy of the findings.

## Results

**Figure 1:**
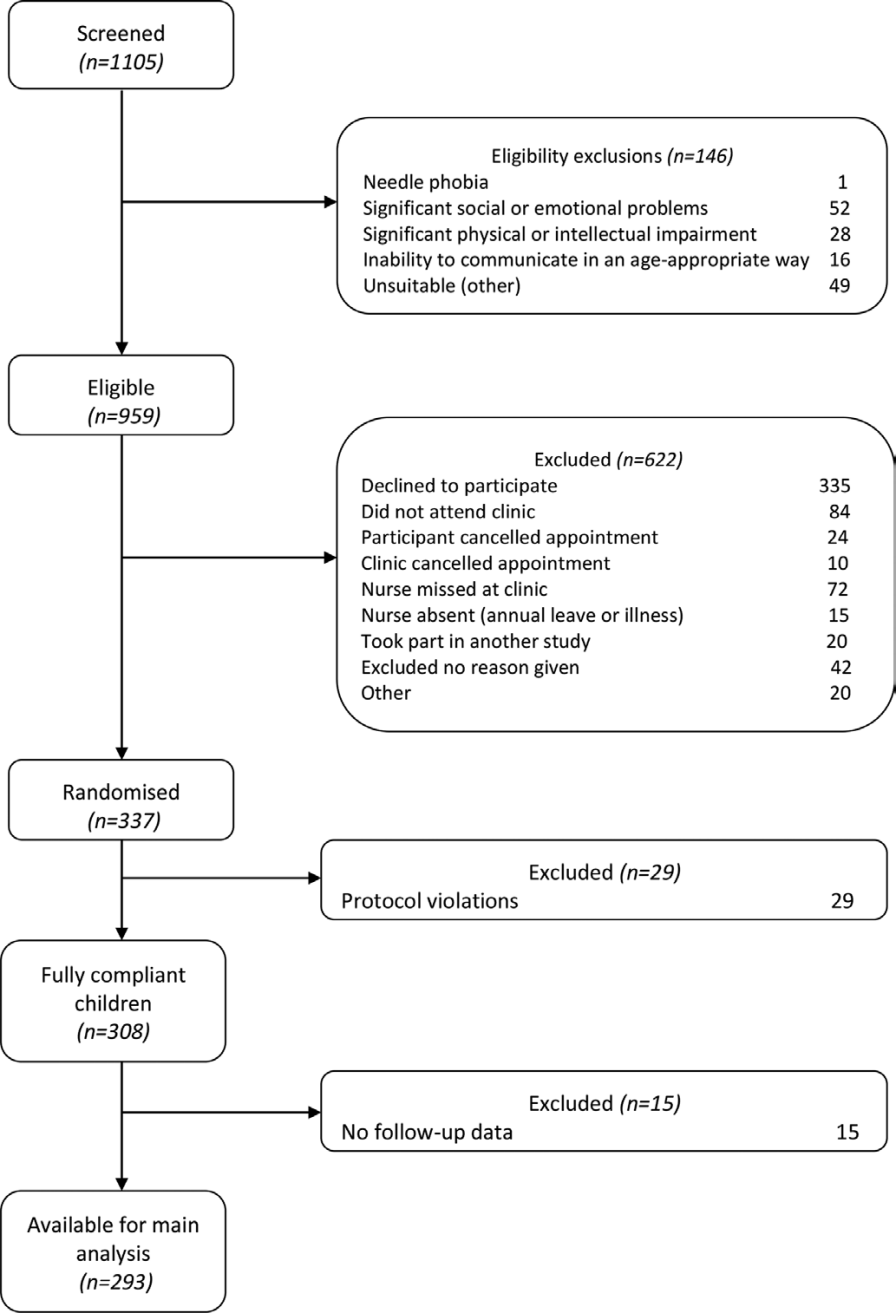
Consolidated Standards of Reporting Trials diagram from screening to analysis.

**Figure 2:**
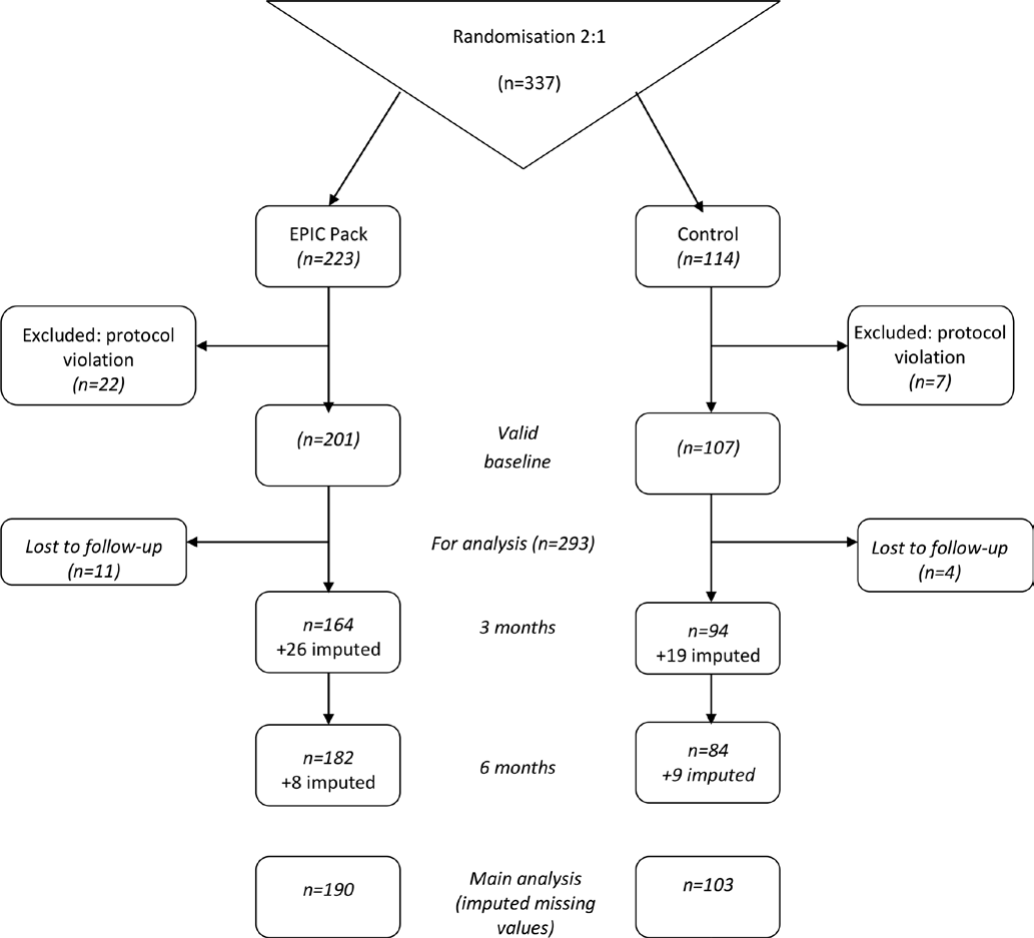
Consolidated Standards of Reporting Trials diagram, showing missing data from screening to analysis. EPIC, Evidence into Practice—Information Counts.

Figures 1 and 2 show the flow of participants through the study. We screened 1105 children identified as potentially eligible from the clinic lists of 11 participating hospitals: 146 (13.2%) were not eligible by the trial criteria, 335 (30.3%) declined to participate after receiving letters of invitation and trial information sheets, 287 (26.0%) were missed in clinic or did not join the trial for other reasons. Hence, we randomised 337 children.

### Response rates

Two protocol violations affected 29 children. More importantly, 21 intervention and seven comparator children did not complete baseline questionnaires before receiving treatment within the trial, most because one centre allowed them to take questionnaires home for return by post. The other violation randomised the same child twice following a change of web servers at another site. Fortunately, sensitivity analysis including these 29 children showed essentially the same results.^15^Figure 2 shows that, of the 308 fully compliant children, 256 (84%) returned questionnaires at 3 months, 266 (86%) did so at 6 months and imputation enabled us to analyse 293 (95%). Thus, the incentive of shopping vouchers achieved much higher response rates than our targets. Hence, the trial was better powered than planned.

### Baseline characteristics

Table 1 shows characteristics of the 293 analysable participants at baseline.

**Table 1:** Characteristics of participants at baseline by allocated group

N (row %)		EPIC kit	Treatment as usual
		190 (65%)	103 (35%)
Demographic characteristics (n (%) unless specified)			
Gender	Male	85 (45)	49 (48)
	Female	105 (55)	54 (52)
Age in years	Range	6.3–18.9	6.4–18.4
	Mean (SD)	12.4 (3.0)	12.7 (3.2)
Ethnicity	White British	179 (94)	101 (98)
	Other	11 (6)	2 (2)
Education and employment	Secondary school	13 (42)	7 (33)
	Further education college	13 (42)	12 (57)
	Other	5 (16)	1 (5)
	Age <16 so not asked	159	86
Living situation	Owner occupied house/flat	156 (82)	86 (83)
	Privately rented house/flat	20 (11)	6 (6)
	Housing assoc./local authority	14 (7)	11 (11)
Years since diagnosis	Range	0.8–16.7	1.2–15.7
	Mean (SD)	7.4 (3.8)	8.0 (3.9)
Type of insulin	Injections	167 (88)	87 (84)
Administration	Pump	23 (12)	16 (16)
Insulin regimen	Once a day	2 (1)	–
	2 times a day	41 (25)	19 (22)
	3 times a day	14 (8)	10 (11)
	4 times a day	95 (57)	45 (52)
	Other (at least 5 times a day)	15 (9)	13 (15)
Blood glucose tests	None	1 (1)	1 (1)
	Once a day	4 (2)	1 (1)
	2 times a day	7 (4)	5 (5)
	3 times a day	31 (16)	16 (16)
	4 times a day	89 (47)	50 (49)
	Other (at least 5 times a day)	57 (30)	29 (28)
	Missing	1	1
HbA1c (%) (mmol/mol) 1	Range	5.9–14.0; 41.0–129.5	6.0–13.7; 42.1–126.2
	Mean	8.77/72.3	8.59/70.4
QoL baseline measures at trial entry			
Child self-report	PedsQL: diabetes module (total scale score 100)	73.63 (14.68)	73.29 (12.17)
	PedsQL: generic module (total scale score 100)	83.70 (12.36)	81.78 (12.63)
	EQ-5D utility score (total scale score 1)	0.9012 (0.1501)	0.8976 (0.1537)
	EQ-5D VAS (total scale score 100)	83.22 (16.98)	77.86 (18.89)
Parent proxy	PedsQL: diabetes module (total scale score 100)	65.82 (15.35)	65.65 (14.02)
	PedsQL: generic module (total scale score 100)	77.86 (14.66)	77.78 (14.43)
	EQ-5D utility score (total scale score 1)	0.8499 (0.1733)	0.8231 (0.1800)
	EQ-5D VAS (total scale score100)	83.02 (16.40)	79.96 (19.11)

EPIC, Evidence into Practice—Information Counts; QoL, quality of life; VAS, Visual analogue scale.

### Primary outcome

Tables 2 and 3 show that, of the five dimensions of the Diabetes PedsQL, only Worry showed adjusted scores significantly favouring self-management kits at 3 months: the mean child-reported difference was +5.87 with SE of 2.19, generating statistical significance level (p) of 0.8% and a 95% CI from +1.57 to +10.18. At 6 months, however, only Treatment Adherence achieved significance—in favour of treatment as usual: the mean child-reported difference was –4.68 with SE of 1.74, generating another p of 0.8% and 95% CI from –8.10 to –1.25. Even worse, intervention children reported significant adverse changes between 3 and 6 months on four of the five Diabetes PedsQL dimensions and thus on the total score: the mean child-reported difference was –3.20 with statistical significance level of 2.0% and 95% CI from –5.73 to –0.67.

**Table 2:** Mixed models adjusted by stratification variables and baseline values by treatment allocated

Outcome variable	Difference (Epic kit –treatment as usual)					Covariates/cofactors sig. at 5%		
	F(1,290)	P value	Mean	SE	95% CI		F(1,290)	P value
**Child self-report**								
PedsQL: general								
Total score	0.76	0.384	−0.96	1.10	−3.13 to 1.21	Baseline	275	<0.001
						Time-point	4.36	0.038
						Treatment group by time-point	5.13	0.024
Physical functioning	0.00	0.974	0.04	1.32	−2.56 to 2.65	Baseline	103	<0.001
						Time-point	5.38	0.021
						Treatment group by time-point	8.14	0.005
Emotional functioning	0.08	0.780	−0.51	1.83	−4.12 to 3.09	Baseline	184	<0.001
Social functioning	0.00	0.971	−0.05	1.46	−2.92 to 2.81	Baseline	181	<0.001
School functioning	4.26	0.040	−2.90	1.40	−5.66 to −0.14	Baseline	271	<0.001
						Treatment group by time-point	9.59	0.002
PedsQL: diabetes								
Total score	0.07	0.798	−0.32	1.26	−2.80 to 2.16	Baseline	183	<0.001
						Treatment group by time-point	5.44	0.020
Diabetes symptoms	0.00	0.955	−0.09	1.55	−3.14 to 2.96	Baseline	170	<0.001
Treatment barriers	0.02	0.876	−0.27	1.71	−3.64 to 3.10	Baseline	174	<0.001
						Treatment group by time-point	4.19	0.042
Treatment adherence	2.60	0.108	−2.38	1.47	−5.28 to 0.52	Baseline	78	<0.001
						Treatment group by time-point	6.87	0.009
Worry	2.76	0.098	3.23	1.94	−0.60 to 7.05	Baseline	176	<0.001
						Treatment group by time-point	4.88	0.028
Communication	0.13	0.720	0.69	1.93	−3.10 to 4.48	Baseline	107	<0.001
						Treatment group by time-point	7.17	0.008
EQ-5D	0.00	0.960	0.001	0.018	−0.034 to.036	Baseline	115	<0.001
						Gender	6.12	0.014
						Length of time since diagnosis	6.82	0.009
EQ-5D: VAS	0.42	0.520	1.10	1.70	−2.26 to 4.45	Baseline	125	<0.001
						Age	4.05	0.045
**Parent proxy**								
PedsQL: general								
Total score	0.66	0.417	0.94	1.15	−1.33 to 3.20	Baseline	264	<0.001
Physical functioning	2.61	0.107	2.22	1.38	−0.48 to 4.93	Baseline	94	<0.001
Emotional functioning	0.06	0.799	−0.48	1.86	−4.15 to 3.20	Baseline	143	<0.001
Social functioning	0.25	0.620	0.72	1.45	−2.13 to 3.57	Baseline	292	<0.001
						Age	5.28	0.022
School functioning	0.463	0.497	0.97	1.42	−1.84 to 3.77	Baseline	316	<0.001
						Time point	6.81	0.010
PedsQL: diabetes								
Total score	2.36	0.125	−1.68	1.09	−3.82 to 0.47	Baseline	412	<0.001
Diabetes symptoms	1.44	0.232	−1.55	1.29	−4.09 to 1.00	Baseline	390	<0.001
Treatment barriers	0.57	0.452	−1.20	1.60	−4.36 to 1.95	Baseline	270	<0.001
						Age	5.67	0.018
Treatment adherence	6.15	0.014	−3.48	1.40	−6.23 to −0.72	Baseline	218	<0.001
Worry	0.15	0.697	−0.77	1.98	−4.66 to 3.12	Baseline	200	<0.001
						Time-point	9.49	0.002
Communication	0.11	0.742	0.67	2.04	−3.34 to 4.69	Baseline	171	<0.001
EQ-5D	1.82	0.178	−0.025	0.019	−0.062 to 0.012	Baseline	71	<0.001
EQ-5D: VAS	0.76	0.385	−1.34	1.54	−4.38 to 1.69	Baseline	171	<0.001
						Gender	5.22	0.023
HbA1c								
mmol/mol	0.11	0.740	−0.40	1.22	−2.80 to 1.99	Baseline	330	<0.001

EPIC, Evidence into Practice—Information Counts; VAS, Visual analogue scale.

**Table 3:** Mixed models: mean effect at 3 and 6 months estimated from main model

Outcome variable	Time point	Difference (Epic kit—treatment as usual)					
		Mean	SE	95% CI	P value	Change (6 months to 3 months)	P value
Child self-report							
PedsQL: general							
Total score	3 months	0.52	1.26	(−1.96 to 2.99)	0.682	−2.96	0.024
	6 months	−2.44	1.30	(−5.01 to 0.13)	0.063		
Physical functioning	3 months	2.16	1.59	(−0.96 to 5.29)	0.174	−4.24	0.005
	6 months	−2.08	1.44	(−4.92 to 0.76)	0.151		
Emotional functioning	3 months	−0.60	2.06	(−4.65 to 3.45)	0.769	0.18	0.932
	6 months	−0.42	2.19	(−4.73 to 3.89)	0.848		
Social functioning	3 months	0.55	1.62	(−2.64 to 3.75)	0.734	−1.21	0.455
	6 months	−0.66	1.72	(−4.03 to 2.72)	0.701		
School functioning	3 months	−0.01	1.63	(−3.21 to 3.20)	0.997	−5.78	0.002
	6 months	−5.79	1.74	(−9.21 to −2.36)	0.001		
PedsQL: diabetes							
Total score	3 months	1.23	1.39	(−1.50 to 3.96)	0.375	−3.11	0.020
	6 months	−1.88	1.46	(−4.76 to 1.00)	0.200		
Diabetes symptoms	3 months	0.14	1.72	(−3.26 to 3.53)	0.938	−0.45	0.803
	6 months	−0.31	1.81	(−3.88 to 3.26)	0.865		
Treatment barriers	3 months	1.77	1.91	(−1.98 to 5.52)	0.355	−4.07	0.042
	6 months	−2.30	2.05	(−6.34 to 1.74)	0.263		
Treatment adherence	3 months	−0.08	1.69	(−3.40 to 3.25)	0.964	−4.76	0.009
	6 months	−4.68	1.74	(−8.10 to −1.25)	0.008		
Worry	3 months	5.87	2.19	(1.57 to 10.18)	0.008	−5.29	0.028
	6 months	0.58	2.37	(−4.09 to 5.25)	0.808		
Communication	3 months	3.66	2.10	(−0.47 to 7.80)	0.082	−5.94	0.008
	6 months	−2.28	2.34	(−6.89 to 2.32)	0.330		
EQ-5D	3 months	0.013	0.023	(−0.032 to 0.059)	0.568	−0.025	0.304
	6 months	−0.011	0.019	(−0.050 to 0.027)	0.555		
							
EQ-5D: VAS	3 months	0.58	1.83	(−3.02 to 4.18)	0.751	1.04	0.569
	6 months	1.62	2.03	(−2.37 to 5.60)	0.426		
							
Parent proxy							
PedsQL: general							
Total score	3 months	0.04	0.976	(−2.43 to 2.51)	0.976	1.79	0.114
	6 months	1.83	0.191	(−0.92 to 4.58)	0.191		
Physical functioning	3 months	1.53	1.53	(−1.49 to 4.55)	0.319	1.39	0.399
	6 months	2.92	1.66	(−0.36 to 6.19)	0.081		
Emotional functioning	3 months	−2.44	2.10	(−6.58 to 1.71)	0.248	3.93	0.068
	6 months	1.49	2.20	(−2.84 to 5.81)	0.499		
Social functioning	3 months	1.16	1.60	(−1.99 to 4.30)	0.470	−0.88	0.586
	6 months	0.28	1.71	(−3.09 to 3.65)	0.870		
School functioning	3 months	−0.53	1.72	(−3.91 to 2.85)	0.757	3.00	0.114
	6 months	2.47	1.70	(−0.88 to 5.82)	0.148		
PedsQL: diabetes							
Total score	3 months	−2.29	1.20	(−4.64 to 0.07)	0.057	3.49	0.781
	6 months	1.20	1.38	(−3.78 to 1.65)	0.440		
Diabetes symptoms	3 months	−2.93	1.40	(−5.68 to −0.19)	0.036	2.77	0.074
	6 months	−0.16	1.61	(−3.33 to 3.01)	0.920		
Treatment barriers	3 months	−2.71	1.85	(−6.36 to 0.94)	0.145	3.01	0.145
	6 months	0.30	1.98	(−3.59 to 4.19)	0.880		
Treatment adherence	3 months	−3.86	1.72	(−7.25 to −0.47)	0.026	0.77	0.702
	6 months	−3.09	1.73	(−6.50 to 0.32)	0.076		
Worry	3 months	1.15	2.44	(−3.64 to 5.94)	0.637	3.84	0.161
	6 months	−2.69	2.38	(−7.37 to 1.98)	0.258		
Communication	3 months	1.04	2.43	(−3.74 to 5.83)	0.668	1.98	0.768
	6 months	3.02	2.36	(−4.34 to 4.94)	0.898		
EQ-5D	3 months	−0.024	0.023	(−0.069 to 0.020)	0.280	−0.001	0.963
	6 months	−0.026	0.022	(−0.070 to 0.019)	0.254		
EQ-5D: VAS	3 months	−2.00	1.78	(−5.50 to 1.51)	0.263	1.31	0.484
	6 months	−0.69	1.82	(−4.28 to 2.90)	0.706		
HbA1c							
mmol/mol	3 months	−0.63	1.36	(−3.31 to 2.04)	0.641	0.45	0.765
	6 months	−0.19	1.46	(−3.07 to 2.70)	0.899		

EPIC, Evidence into Practice—Information Counts; VAS, Visual analogue scale.

### Secondary outcomes

Moreover, there was no evidence of change in HbA1c. Participants started the trial with a mean baseline HbA1c of 72 mmol/mol. Whether adjusted by baseline scores and stratification variables or not, this mean remained virtually unchanged during the follow-up period: after 3 months self-managing children had reduced their adjusted HbA1c by only –0.63 mmol/mol relative to comparator children (p=0.64; 95% CI from –3.31 to +2.04); after 6 months the reduction was only –0.19 mmol/mol (p=0.90; 95% CI from –3.07 to +2.70). At baseline, only 16% of children across both treatment groups had achieved HbA1c levels ≤58 mmol/mol, and at 6 months only 18%, thereby matching the national average in 2012/13.^5^

Tables 2 and 3 also show that the only statistically significant difference between groups in General PedsQL or EQ-5D-3L was that self-managing children reported that their School Functioning after 6 months was worse by –5.79 on average (p<0.001; 95% CI from –9.21 to –2.36). Those intervention children also reported significant adverse changes between 3 and 6 months on two of the four Diabetes PedsQL dimensions and thus on the total score: the mean child-reported difference was –5.78 (p=0.002; 95% CI from –9.39 to –2.17).

However, the parent-reported PedsQL scores showed a different pattern from the child-reported scores: no delayed negative effects were apparent; instead, comparator children were significantly better after 3 months in Diabetes Symptoms by –2.93 (p=0.036; 95% CI from –5.68 to –0.19); and in Treatment Adherence by –3.86 (p=0.026; 95% CI from –7.25 to –0.47).

### Adverse events

Participating clinicians reported 31 SAEs to the trial team—22 in the intervention group and nine in the comparator group, yielding a relative risk of 1.33 (p=0.45; 95% CI from 0.63 to 2.77). The CPI, and the Chairs of the Data Monitoring and Ethics Committee and the Trial Steering Committee, reviewed and confirmed these SAEs. The CPI and research team judged that four SAEs from the intervention group and two from the Control Group were SARs possibly related to the EPIC intervention or to treatment as usual, yielding a relative risk of 1.08 (p=0.92; 95% CI from 0.20 to 5.82).

### Sensitivity analyses

Our sensitivity analysis confirmed that participants with incomplete outcomes (withdrawals or those lost to follow-up) did not change the findings of this trial.

### Posthoc analysis

Given the shortage of treatment effects and the varying size of the three age subgroups, we undertook posthoc analysis to see if there was any evidence of an effect within individual age groups bands. We found no evidence of differences in outcomes by age group.

### Cost consequences

We based economic analysis on 233 (80%) of the 293 children in the effectiveness analysis. We excluded 60 (20%) children with incomplete data on costs and service use because we could not be sure that these were missing at random. The mean total intervention unit cost of producing and administering intervention kits was £185 (table 4A). The mean total cost (NHS costs including intervention kit and administration costs) was £136 (bootstrapped 95% CI: -£52 to £296) higher for the intervention group than for the comparator group, but this difference was not statistically significant (table 4B). We undertook bootstrapping with 1000 replicates to estimate a 95% CI around this mean difference in costs and consequences between groups. For consequences, there were no significant mean differences for any outcome. Sensitivity analysis postulating that consultants instead of PDSNs see children in clinic but for the same 13.2 min, increased the mean difference in total costs of service use between groups from £136 to £182 (bootstrapped 95% CI: -£9 to £339), but this difference was still not statistically significant.

**Table 4A:** Costs of producing and distributing EPIC self-management intervention kits and distributing them to participants by PDSNs in clinics in 2011 UK pounds (£)

Age group	6–10 years		11–15 years		16–18 years		Across all ages
Administration route	Injection	Pump	Injection	Pump	Injection	Pump	
Cost of self-management kit* (£)	11.57	16.67	12.09	17.19	22.07	28.29	
Cost of extra PDSN appointments† (£)	166.66	166.66	166.66	166.66	166.66	166.66	
**Total cost per participant (£)**	**178.23**	**183.33**	**178.75**	**183.85**	**188.73**	**194.95**	**184.64**
Number in intervention group	49	9	66	8	21	5	158
**Total cost of intervention (£)**	**8733**	**1650**	**11 798**	**1471**	**3963**	**975**	**28 590**
Average cost per participant (£)							**180.95**

Bold figures represent total costs.

*Including ‘treatment cost’ of producing and printing age-specific diabetes diaries, but not ‘research cost’ of developing these diaries.

†Including ‘treatment cost’ of estimated time of Paediatric Diabetes Specialist Nurses (PDSNs) in teaching children about the kit, but not ‘research cost’ of research nurses distributing the kit.

**Table 4B:** Health service use costs and consequences by allocated group over 6 months

	Intervention kits (n=158) Mean (SD)	Treatment as usual (n=75) Mean (SD)	Intervention minus treatment as usual (bootstrapped 95% CIs)
*Costs*			
Primary care (£)*	67 (111)	61 (90)	6 (−22 to 32)
Secondary care (£)*	454 (524)	504 (648)	−50 (−226 to 103)
EPIC intervention cost (£)*†	181 (4)	0 (0)	181
Total cost/participant (£)	702 (558)	566 (664)	136 (−52 to 296)
*Consequences*			
*Participant self-report*			
QALYs	0.446 (0.0741)	0.447 (0.0784)	−0.001 (−0.0209 to 0.0189)
*Parent-proxy*			
QALYs	0.415 (0.0785)	0.418 (0.0831)	−0.003 (−0.0238 to 0.0188)

*Mean (SD) total cost per participant (£).

†Cost of intervention includes the ‘treatment costs’ of producing and distributing the kit to participants by Paediatric Diabetes Specialist Nurses in clinics; those of producing and distributing the intervention diabetes diary; but not the ‘research costs’ of developing kit or diary.

EPIC, Evidence into Practice—Information Counts; QALYs, Quality Adjusted Life Years.

### Process evaluation

We present key findings from the process evaluation^12^ to illustrate the wider context within which children were initially excited but thereafter did not engage as intended with diabetes self-management generally or use the standardised kits specifically. We offer an explanation as to how the context was created for this mechanism to occur.

#### Normalisation in children’s self-management and relationships with professionals

After initial excitement, most children said that they did not use the EPIC kits as intended; a few rejected them completely and put them out of sight, for example in the loft. Analysis of the words, messages and images in children’s self-management information found that they generally presented rules to manage diabetes supported by images of being ‘normal’ like other children if they followed those rules and did what professionals said. The process evaluation showed that these authoritarian normalisation messages did not always resonate with children, especially teenagers, as they did not feel normal because their life was frequently defined by diabetes, which they disliked. The presence of the EPIC kits and the messages they contained caused increasing levels of worry and anxiety. So they tried to hide diabetes by not making ‘self-management’ visible to themselves or others. Hence, few children took a diabetes diary to school or wanted to test their blood sugar levels.

#### Children’s inability to associate blood glucose tests with better management

Baseline questionnaires from 308 children and their parents at entry to the trial show that they knew how many times a day they should record their blood glucose. In reality, most children, especially teenagers and irrespective of allocation, did not use or even see the need to record or observe trends in blood glucose levels to titrate their insulin dose. Of those interviewed in the intervention treatment group, around half of 6–10 year olds, less than half of 11–15 year olds, but only around 20% of 16–18 year olds said that they or their parents recorded blood glucose levels; fewer still appeared to use levels to titrate insulin doses. Children more commonly neglected the age-appropriate self-management information provided. Many children thought that they were recording this information for the benefit of diabetes professionals and made no link between blood glucose testing and gaining better diabetes control.

#### Children’s ignorance of risky behaviour and long-term complications of diabetes

By not titrating insulin doses to blood glucose levels, many children took risks with their diabetes-related health; many teenagers appeared unconcerned about the potential consequences. Some parents said they wanted to protect their children from receiving information on risks and complications, whereas others wanted to expose their children to the reality of life threatening complications like renal failure. Discourse analysis showed that children’s diabetes information resources for ages 6–10 years rarely mentioned risks or complications of poorly controlled diabetes; while those for ages 11–15 years were usually vague about serious risks and long-term complications of poorly controlled diabetes. In contrast, information distributed on entry to adult diabetes services was explicit about risks of long-term complications and the resulting need for self-management to minimise these.

#### Promotion of intervention by diabetes professionals and parents

Most diabetes teams did not actively engage with the intervention kits or encourage their use in routine consultations. Around a third of children approached declined to participate. Often, there were modifications to intended intervention delivery; for example the research nurse, not a member of the child’s diabetes team, gave the kit and diary to the child. There was little individualisation or tailoring of intervention kits. Around half the PDSNs regulated the information given to children and knowingly withheld or removed information on lifestyle issues and risks of complications before distribution to children. From the kit for ages 11–15 distributed to 103 participants, professionals reported removing several topics as inappropriate: sex and beyond (12 times), drinking alcohol (11 times), body piercing (eight times) and carbohydrate awareness (four times). After children had received the kits, many parents had removed anything they thought unsuitable, notably the ‘lifestyle’ resources, if not already removed by the PDSN.

## Discussion

### Principal findings

This pragmatic trial found no evidence of benefit from age-appropriate diabetes self-management EPIC kits for children. Diabetes eduaction resources given to the Treatment as usual group varied widely and many had not recceived any for several years since diagnosis, and is described in more detail elsewhere. ^12^ Of the five dimensions of the Diabetes PedsQL, Worry showed adjusted scores significantly favouring self-management kits at 3 months but Treatment Adherence significantly favoured controls at 6 months. Furthermore, children using EPIC self-management kits reported significantly worse changes between 3 and 6 months on four of the five Diabetes PedsQL dimensions and thus on the total score. There was no evidence of change in HbA1c; only 18% of participants in each group achieved recommended levels at 6 months. This was apparently because the EPIC kits alienated children and parents, and their use of kits and recording of blood glucose was poor. Moreover, five^29–33^ other contemporaneous UK trials of educational interventions for childhood diabetes reported no benefit and little rapport between children, their parents, clinic staff and the interventions. The other five interventions comprised structured diabetes education, family-based diabetes education and support^30–33^ and training in communication skills.^29^ None of these six UK trials (including EPIC) showed any difference in HbA1c between groups; or evidence of any other benefit. EPIC is the first trial to make clear a deleterious intervention effect over time. As fewer than 20% of all 2018 children across the six trials achieved glycaemic control (pre-2015 HbA1c target of ≤58 mmol/mol), this meant that over 80% of children were at risk of serious complications. All six trials reported concerns about intervention fidelity. Attendance at additional diabetes teaching sessions was highly variable, and those with the highest HbA1c were least likely to attend. A recent systematic review of 10 trials of education and psychoeducation interventions (including the five trials above but excluding EPIC as HbA1c had not been fully analysed at the time) showed a non-significant reduction in HbA1c attributable to the intervention (pooled standardized mean difference (SMD)=−0.06, 95% CI: −0.21 to 0.09).^34^

Our parallel analysis of the words and images in diabetes resources provides new insights into the potential source of the surprisingly adverse effects of the kits. These stem in part from children’s rejection of ‘unwelcome’ information which labelled them as different in having diabetes and authoritarian instructions like ‘take me with you wherever you go’. More generally, the discourse of ‘normalisation’ through optimal management and insulin as a social enabler appears counterproductive in promoting desired behaviour change. Poor relationships between children, parents and diabetes professionals is another reason for lack of ‘compliance’ with diabetes professionals’ expectations. In short, diabetes communicators do not yet know how to convey effective messages to children. One trial (DEPICTED),^29^ which attempted to train healthcare professionals do this, also failed to show any benefit.

### Strengths and weaknesses of this study

We based the EPIC kit on extensive research with children, parents and professionals; and met international standards for patient information.^12 13^ We powered the EPIC trial to detect plausible effect sizes for the entire age-range, and surpassed recruitment targets.^20 21^ Particular strengths included the preparatory discourse analysis of children’s diabetes self-management information and the process evaluation in which children assessed the ways in which they received key diabetes self-management information. In retrospect, the major weakness was the unpopularity of the EPIC kit.

### Interpretation

Lack of progress in meeting the NICE target for HbA1c in children stimulated the commissioning of this and five other contemporaneous trials by UK funders to test various approaches to promote optimal self-management. Other promising developments since the delivery of this trial include electronic management systems, diabetes phone and iPad applications, and personalised web-based diabetes training programmes.^27–30^ However, these are unlikely to benefit children if, despite receiving good information, they still do not make the link between blood glucose testing and achieving glycaemic control through insulin, diet and lifestyle management.

In contrast, international studies show that children can achieve acceptable glycaemic control through investment in structured education and patient management following diagnosis, with outcomes ranging from 29% to over 50% of children achieving an HbA1c level less than ≤58 mmol/mol^35 36^ this is much higher than the 18% achieved by EPIC and the UK in general. The Hvidøre Study Group^35^ identified management from the diagnosis of the disease, positive and shared attitudes within diabetes teams and greater patient empowerment as factors that enhanced glycaemic control. Though we understand these factors, we do not know why UK children cannot achieve similar standards.

### Implications for practice and research

Our process evaluation and discourse analysis identified two problems which have not yet been adequately addressed. First, we need to understand better how children respond to ‘authority’ in relationships with diabetes professionals. Expected regular attendance at outpatient clinics, the normative nature of most self-management information, and the withholding of requested lifestyle information all reinforce the power imbalance. Despite the intended focus on ‘normalisation’, children feel different, dislike being different and tend to reject any intervention that reminds them of that difference. The second problem is that children, parents and professionals cannot reconcile clinical risk definitions and personal ones. Children assess risks differently from diabetes professionals and take rational decisions about what is acceptable for them. To overcome this, professionals may need to address risk collaboratively rather than hierarchically and this would require a complete rethink as to how diabetes services are designed and delivered. Motivational interviewing has, for example, been shown to be an effective method of facilitating behavioural changes in teenagers with type 1 diabetes with subsequent improvement in their glycaemic control.^37^

There are also implications for trial methods and conduct. First, we need to encourage research and clinical teams to implement complex interventions like this rigorously but also flexibly. Second, as the care of children’s diabetes varies widely across the UK, there is merit in engaging the diabetes teams likely to contribute to a planned trial in design and planning.

## Supplementary Material

Reviewer comments

Author's manuscript
