# Supplementary material for: Standardised self-management kits for children with type 1 diabetes: pragmatic randomised trial of effectiveness and cost-effectiveness
Source: BMJ Open. 2020 Mar 12;10(3):e032163. doi: 10.1136/bmjopen-2019-032163 (PMC7069268; doi:10.1136/bmjopen-2019-032163)
Supplement: Author's manuscript [file bmjopen-2019-032163.draft_revisions.pdf]

BMJ Open is committed to open peer review. As part of this commitment we make the peer review history of every article we publish publicly available.

When an article is published we post the peer reviewers' comments and the authors' responses online. We also post the versions of the paper that were used during peer review. These are the versions that the peer review comments apply to.

The versions of the paper that follow are the versions that were submitted during the peer review process. They are not the versions of record or the final published versions. They should not be cited or distributed as the published version of this manuscript.

BMJ Open is an open access journal and the full, final, typeset and author-corrected version of record of the manuscript is available on our site with no access controls, subscription charges or pay-per-view fees (http://bmjopen.bmj.com).

If you have any questions on BMJ Open's open peer review process please email info.bmjopen@bmj.com

BMJ Open

Standardised self-management kits for children with type 1 diabetes: pragmatic randomised trial of effectiveness and cost-effectiveness

Journal:	*BMJ Open*
Manuscript ID	bmjopen-2019-032163
Article Type:	Original research
Date Submitted by the Author:	05-Jun-2019
Complete List of Authors:	Noyes, Jane; Bangor University, School of Social Sciences<br>Allen, Davina; Cardiff University<br>Carter, Cynthia; Cardiff University, School of Journalism, Media and Culture<br>Edwards, Deborah; Cardiff University, School of Healthcare Sciences<br>Edwards, Rhiannon; Bangor University, Centre for Health Economics & Medicines Evaluation<br>Russell, Daphne; Swansea University, College of Medicine<br>Russell, Ian; Swansea University, Medicine<br>Spencer, Llinos; Bangor University, Centre for Health Economics and Medicine Evaluation<br>Sylvestre, Yvonne; University of Manchester , Manchester Clinical Trials Unit<br>Whitaker, Rhiannon; Whitaker Research Ltd. ,<br>Yeo, S. T.; University of Bangor School of Healthcare Sciences, Centre for Health Economics and Medicines Evaluation (CHEME)<br>Gregory, John; Cardiff University, School of Medicine
Keywords:	Paediatric endocrinology < DIABETES & ENDOCRINOLOGY, HEALTH SERVICES ADMINISTRATION & MANAGEMENT, Health economics < HEALTH SERVICES ADMINISTRATION & MANAGEMENT

SCHOLARONE™
Manuscripts

I, the Submitting Author has the right to grant and does grant on behalf of all authors of the Work (as defined in the below author licence), an exclusive licence and/or a non-exclusive licence for contributions from authors who are: i) UK Crown employees; ii) where BMJ has agreed a CC-BY licence shall apply, and/or iii) in accordance with the terms applicable for US Federal Government officers or employees acting as part of their official duties; on a worldwide, perpetual, irrevocable, royalty-free basis to BMJ Publishing Group Ltd ("BMJ") its licensees and where the relevant Journal is co-owned by BMJ to the co-owners of the Journal, to publish the Work in this journal and any other BMJ products and to exploit all rights, as set out in our licence.

The Submitting Author accepts and understands that any supply made under these terms is made by BMJ to the Submitting Author unless you are acting as an employee on behalf of your employer or a postgraduate student of an affiliated institution which is paying any applicable article publishing charge ("APC") for Open Access articles. Where the Submitting Author wishes to make the Work available on an Open Access basis (and intends to pay the relevant APC), the terms of reuse of such Open Access shall be governed by a Creative Commons licence – details of these licences and which Creative Commons licence will apply to this Work are set out in our licence referred to above.

Other than as permitted in any relevant BMJ Author's Self Archiving Policies, I confirm this Work has not been accepted for publication elsewhere, is not being considered for publication elsewhere and does not duplicate material already published. I confirm all authors consent to publication of this Work and authorise the granting of this licence.

For peer review only

TITLE PAGE

Standardised self-management kits for children with type 1 diabetes: pragmatic randomised trial of effectiveness and cost-effectiveness

Corresponding Author: Jane Noyes,
Address: Neuadd Ogwen, School of Social Sciences, Bangor University, Wales, LL57 2DG, UK
Email: jane.noyes@bangor.ac.uk
Phone:+44 (0) 1248 388519
Twitter: @Jane_Noyes
Orchid: https://orcid.org/0000-0003-4238-5984

Co-authors:
Davina Allen, Professor of Nursing, School of Healthcare Sciences, Cardiff University, Eastgate House, Newport Road, Cardiff, United Kingdom, CF24 0AB, Email: allenda@cardiff.ac.uk

Cynthia Carter, Reader, School of Journalism, Media and Culture
Cardiff University, Two Central Square, Cardiff, Wales, UK CF10 1FS
email:cartercl@cardiff.ac.uk Orcid: https://orcid.org/0000-0002-5424-9835

Deborah Edwards, Research Associate, School of Healthcare Sciences, Cardiff University, Eastgate House, 35-43 Newport Road / Cardiff / CF24 0AB
Email: edwardsdj@cardiff.ac.uk. https://orcid.org/0000-0003-1885-9297

Rhiannon T Edwards, Co- Director Centre for Health Economics and Medicines Evaluation, Ardudwy Hall, Bangor University, Bangor, Gwynedd, LL57 2PZ, Email: r.t.edwards@bangor.ac.uk

Daphne Russell, Senior Trial Statistician, Swansea University Medical School, Singleton Park Swansea, SA2 8PP. E-mail: daphne.russell@cantab.net

Ian T Russell, Emeritus Professor of Clinical Trials, Swansea University Medical School, Singleton Park, Swansea, SA2 8PP i.t.russell@swansea.ac.uk

Llinos H Spencer, Research Officer, Centre for Health Economics and Medicines Evaluation, Bangor University, Neuadd Ardudwy, Normal Site, Bangor, Gwynedd, LL57 2PZ
Email: l.spencer@bangor.ac.uk. Orchid: https://orcid.org/0000-0002-7075-8015

Yvonne Sylvestre, Statistician, Christie Hospital NHS Trust, Manchester, UK. Email: ysylvestre@gmail.com

Rhiannon Whitaker, Statistician, Whitaker Research Ltd, 8 Whitehall Road, Rhos on Sea, LL28 4HW. Email: rhiannon@whitres.co.uk

Seow Tien Yeo, Research Officer (Health Economics), Centre for Health Economics and Medicines Evaluation (CHEME), School of Health Sciences, Bangor University, Ardudwy Building, Normal Site, Bangor, Gwynedd, LL57 2PZ. Email: s.t.yeo@bangor.ac.uk Orchid: http://orcid.org/0000-0001-7496-1742

John W Gregory, Professor in Paediatric Endocrinology & Honorary Consultant, Division of Population Medicine, School of Medicine, Cardiff University, Heath Park, Cardiff, CF14 4XN.
Email: wchjwg@Cardiff.ac.uk

For peer review only - http://bmjopen.bmj.com/site/about/guidelines.xhtml

Standardised self-management kits for children with type 1 diabetes: pragmatic randomised trial of effectiveness and cost-effectiveness

Abstract

Objective: To estimate the effectiveness of standardised self-management kits for children with type 1 diabetes.

Design: Pragmatic trial with randomisation ratio of 2 intervention: 1 control. Qualitative process evaluation.

Setting: 11 diabetes clinics in England and Wales.

Participants: Between February 2010 and August 2011 we validly randomised 308 children aged six to 18 years; 201 received the intervention.

Intervention: We designed kits to empower children to achieve glycaemic control, notably by recording blood glucose and titrating insulin. The comparator was usual treatment.

Outcome measures at 3 and 6 months: Primary: Diabetes PedsQL. Secondary: HbA1c; General PedsQL; EQ-5D; healthcare resource use.

Results: Of the five Diabetes PedsQL dimensions, Worry showed adjusted scores significantly favouring self-management kits at three months (mean child-reported difference = +5.87; standard error [SE] = 2.19; 95% confidence interval [CI] from +1.57 to +10.18; p = 0.008); but Treatment Adherence significantly favoured controls at six months (mean child-reported difference = -4.68; SE = 1.74; 95% CI from -8.10 to -1.25; p = 0.008). Intervention children reported significantly worse changes between three and six months on four of the five Diabetes PedsQL dimensions and on the total score (mean difference = -3.20; SE = 1.33; 95% CI from -5.73 to -0.67; p = 0.020). There was no evidence of change in HbA1c; only 18% of participants in each group achieved recommended levels at 6 months. No serious adverse reactions attributable to the intervention or its absence were reported.

Children's use of kits was poor. Few children or parents associated blood glucose readings with better glycaemic control. The kits, costing £185, alienated many children and parents.

Conclusions: Standardised kits showed no evidence of benefit, inhibited self-management and increased worry. Future research should study relationships between children and professionals, and seek new methods of helping children and parents to manage diabetes.

For peer review only - http://bmjopen.bmj.com/site/about/guidelines.xhtml

Trial registration: ISRCTN17551624

Funding: National Institute for Health Research

Strengths and limitations of this study

- The self-management kits that were tested in this trial were designed with children and young people and their parents in a 3 year study.
- The paper reports a fully powered pragmatic trial of children's self-management kits in routine practice.
- Our large process evaluation provided a detailed explanation of the mechanisms that appear to lead to negative outcomes and lack of engagement by children and parents.
- A cost-effectiveness analysis determined the cost of the intervention.

For peer review only - http://bmjopen.bmj.com/site/about/guidelines.xhtml

Introduction

Managing diabetes at all ages costs the National Health Service (NHS) nearly £10 billion a year; 80% of this is for managing avoidable complications.[1] Learning to manage one's diabetes in childhood is important to prevent long-term and potentially life-threatening complications of poor glycaemic control. Diabetes care pathways[2-5] have been available for over a decade and the proportion of children in England and Wales who achieved the previous National Institute for Health and Care Excellence (NICE) target of an HbA_{1c} level of ≤58 mmol/mol has slowly increased from 14.5% in 2009 to 15.8% in 2013 and 26.6% in 2015.[6-8] In 2015 NICE further amended the target to ≤48mmol/mol.[9] Schools vary considerably in the support given to children to manage their insulin administration, diet, and participation in extracurricular activities and sports.[10] Achieving optimal glycaemic control is most difficult during transition from paediatric to adult services when young people become independent of parents and families.[11]

Optimal diabetes self-management requires titration of insulin doses against blood glucose levels, dietary intake and planned physical activities.[3,9] This skill is essential for children to participate fully in school life and social activities outside school.[10] Models of children's diabetes care emphasise a family-centred approach with intensive education and support following diagnosis, with increasing responsibility for care transferred to the child over time.[6-7] There has been no standardisation of diabetes self-management information given to children to use at home and school.

To prepare for the trial, research was undertaken in the current and a previous study with children, young people and parents to identify the types and formats of self-management information likely to inspire behaviour change in children and young people with diabetes.[12,13] Our systematic review of educational and support interventions to improve diabetes self-management in schools revealed no effective interventions, but many barriers to self-management when children were away from their parents.[10] Hence the goal of this trial was to evaluate whether standardised age-appropriate self-management kits motivate children and their families to avoid complications caused by uncontrolled blood sugar levels. Our primary aim was to assess whether the kits enabled children to manage their type 1 diabetes by titrating their insulin dose against regular blood glucose readings. Our secondary aim was to assess how children, their families and diabetes professionals perceived and used these kits.

For peer review only - http://bmjopen.bmj.com/site/about/guidelines.xhtml

The subsequent availability of published reports for five other contemporaneous trials of United Kingdom (UK) children's diabetes education interventions created a new opportunity to review all six trials and explore why none of these six interventions had any effect. In particular, since the original report was pubished in the NIHR journals library[12], we have now reanalysed HbA1c, a secondary outcome measure in our trial so that results can be discussed in relation to five contemporaneous UK trials and other international studies. We have also undertaken a more detailed analysis of the sub-domains of Diabetes PedsQL, the primary outcome measure of our trial, to better understand the benefits and harms of the intervention.

Methods

Study design and participants

We conducted a pragmatic randomised trial, including economic and process evaluations, in NHS paediatric diabetes clinics in England and Wales (ISRCTN 17551624).[12-14] The Wales Multi-Centre Research Ethics Committee gave approval (08/MRE09/57). The Medicines for Children Research Network (England) and the Children and Young People Research Network (Wales) recruited diabetes multi-disciplinary teams in 11 NHS District General Hospitals.

Intervention

Children in the comparator group received treatment as usually provided in each of the 11 diabetes clinics.[14] A record of 'usual care' was made in order to have a clear idea of the comparator with which EPIC was being compared. Children in the intervention group received a standardised but flexible self-management kit known as "Evidence into Practice – Information Counts" (EPIC). Following extensive literature review, consultation and fieldwork,[10,12-14] EPIC comprised:

- Three age-specific (6-10 years, 11-15 or 16-18) diabetes self-management kits comprising booklets, magazines, leaflets, CDs and website links;
- Three corresponding diaries for those using insulin injections*;
- One diary for children using insulin pumps*;
- Sheets for recording carbohydrate intake; and
- Stickers (6-15 years) and marker pens (11-15 years) for children to personalise their folder.

*children could also use blood glucose recordings downloaded from their blood glucose monitors.

For peer review only - http://bmjopen.bmj.com/site/about/guidelines.xhtml

A detailed account of intervention development and the theoretical basis of the EPIC intervention is reported elsewhere.[12] In brief, we designed EPIC so that children and young people had relevant information to self-manage their diabetes (with support from parents for younger children) consistent with relevant clinical guidelines,[2-5] incorporating age-appropriate preferences for information[13] and consistent with clinical practice in the UK NHS. Key features intended to appeal to children and engage them in EPIC included: presentation to and ownership by the child; age-appropriate messages stressing 'top ten tips' for self-management; the invitation 'take me with you wherever you go'; contextual questions about self-management and life-style; integration into routine encounters with the child's multi-disciplinary team, especially the Paediatrician and the Paediatric Diabetes Specialist Nurse (PDSN); scope for professionals and parents to tailor EPIC to each child and review at subsequent appointments; and encouraging the child to record blood glucose and insulin titration every day to share with professionals. We invited diabetes team members to attend training in their hospital which described EPIC, introduced the manual, and suggested how to engage each child, and how to integrate EPIC into routine care.

Random allocation

Between February 2010 and August 2011 we screened diabetes outpatient clinic lists in 11 hospitals for potentially eligible children between 6 and 18 years with type 1 diabetes. We excluded children with communication difficulties, needle phobia, or other impairments judged inconsistent with the trial. We sent invitation letters and age-specific information sheets to families of eligible children. Research nurses independent of both clinical and research teams sought written informed consent to the trial from parents and children over 16 years, or assent from children under 16 years. Consenting parents and their children provided baseline data.

Research nurses then used a secure web-based dynamic randomisation system[15] to allocate children at random between EPIC and treatment as usual, stratified by hospital, age, gender and whether two years had elapsed since diagnosis; the allocation ratio was two intervention participants for every control. These nurses told children's clinical teams of these allocations so they could initiate EPIC at the next consultation. We followed these children for 6 months.

Masking

For peer review only - http://bmjopen.bmj.com/site/about/guidelines.xhtml

Though it was neither desirable nor feasible to blind clinical staff or participants to treatment allocated, we sought to blind assessors. Analysis was undertaken by an independent trial support unit.

Outcome measures

The primary outcome was children's self-efficacy in coping with their diabetes, measured by child and (proxy) parent versions of the Diabetes PedsQL[16] six months after randomisation, with interim scores at three months. The Diabetes PedsQL comprises 33 items (32 for younger children) covering five domains – diabetes, treatment adherence, treatment barriers, communication and worry. The resulting scores lie between zero and 100 with higher scores indicating better coping.

Secondary outcomes comprised: HbA_{1c} measured at routine quarterly clinics; health-related quality of life measured by child and (proxy) parent versions of the General PedsQL;[17] and health-related quality of life measured by the EQ-5D-3L.[18] We used the youth version for children under 16 years, the adult version for those over 16, and parents' proxy scores for all children. Follow-up questionnaires, completed after three and six months in clinic or by post, also sought data on episodes of diabetic ketoacidosis and health service use, especially hospital admissions for acute complications, recorded on a diabetes-specific version of the Client Service Receipt Inventory.[19] We checked health service use against children's hospital notes.

Children and parents also completed baseline questionnaires covering socio-demographic characteristics and the duration and self-management of their diabetes. Children received £10 vouchers for each questionnaire they completed at 3 or 6 months. Non-responders received both telephone and postal reminders after two and four weeks.

We defined serious adverse events (SAEs) as adverse events that, in the judgement of the relevant site Principal Investigator, were lethal, life threatening, resulting in hospital admission, resulting in persistent or significant disability or incapacity, or otherwise medically significant. We defined serious adverse reactions (SARs) as SAEs that, in the judgement of the Clinical Principal Investigator (CPI) and research team, were definitely, probably or possibly related to the EPIC intervention or to treatment as usual.

Sample size

For peer review only - http://bmjopen.bmj.com/site/about/guidelines.xhtml

To yield 80% power of detecting an effect size of 0.4 in the primary outcome of self-efficacy when using a two-sided 5% significance level, we aimed to analyse 202 children, initially by recruiting 252 children – 168 allocated to EPIC and 84 controls, thus allowing for losing 20% of participants to follow up.[20,21] As fewer participants than expected initially responded to questionnaires, we reviewed these calculations in consultation with the Data Monitoring Committee and increased the target to 337 to allow for losing 40% to follow up. We also introduced monetary vouchers for completed questionnaires.

Statistical analyses

Analysis was by treatment allocated. We imputed missing quality of life data in accordance with published guidance for each measure. [22,23] We used the fully conditional specification technique and five multiple imputations across time points to impute these data.[22] We compared differences between treatment groups using mixed models to undertake repeated-measures analysis of variance, adjusting for stratification variables and baseline values. We estimated parameters for three fixed factors – the time-points of three and six months and treatment group. We modelled hospital as a random factor. We included the interaction between treatment group and time-point to test whether differences between treatment groups varied between time-points. These analyses modelled diabetes self-efficacy (Diabetes PedsQL), quality of life (General PedsQL) and health utility (EQ-5D), both to study change in individuals, and in cohort analysis to compare change in group means.

Economic analysis

We costed the age-specific EPIC kits by recording quantities and costs of materials used to produce them, and estimating the mean additional time taken by PDSNs. We collected retrospective data on children's use of primary and secondary healthcare services over the previous three months. We applied national unit costs in 2010-11 pounds sterling (£) to these services.[24,25] As we followed participants for only six months, we did not discount costs or effects.[26] We undertook cost-consequence analysis from an NHS perspective and tested the sensitivity of findings to the substitution of consultants for nurses in presenting the EPIC kits.

Process evaluation

After the trial we recruited a second sample for semi-structured recorded interviews in depth. The process evaluation was conducted up to December 2013. We used purposive sampling to

For peer review only - http://bmjopen.bmj.com/site/about/guidelines.xhtml

generate maximum variation in the ages, genders, times since diagnosis, and types of insulin transfer (injection or pump) of 41 children allocated to EPIC, and 19 comparator children. We also interviewed 66 parents and family members of these children. These interviews explored: views and experiences of both EPIC kits and treatment as usual; how participants managed self-care at home and in school and other social contexts; and children's interactions with diabetes teams. Before the trial we interviewed professionals in each of the 11 hospitals about their previous practice; after the trial we surveyed them by post about how they had implemented EPIC. We recorded interviews, transcribed them verbatim, and analysed them using the thematic framework approach.[27] We mapped the resulting themes onto the underlying theory and developed higher level themes and understanding in depth.

As part of the process evaluation we also undertook a discourse analysis of a purposive sample of children's diabetes resources used in the UK NHS.[28] Discourse analysis is a way of identifying and analysing the assumptions made by information sources about their relationships with their readership, assumptions that had shaped the messages of the selected resources, some of which had appeared in the intervention pack and may also have been available to comparator children.

Patient and public involvement

Children, parents and public representatives were actively involved in an extensive three year prior study, as well as the current study to develop the educational intervention.[12] A core set of diabetes education materials were co-designed by children with type 1 diabetes of various ages. Children and parents were represented in the trial advisory group. All participants received a child-centred copy of the findings.

Results

Figures 1a and 1b show the flow of participants through the study. We screened 1105 children identified as potentially eligible from the clinic lists of 11 participating hospitals: 146 (13.2%) were not eligible by the trial criteria, 335 (30.3%) declined to participate after receiving letters of invitation and trial information sheets, 287 (26.0%) were missed in clinic or did not join the trial for other reasons. Hence we randomised 337 children.

Insert Figures 1a and 1b

For peer review only - http://bmjopen.bmj.com/site/about/guidelines.xhtml

Response rates

Two protocol violations affected 29 children. More importantly 21 intervention and 7 comparator children did not complete baseline questionnaires before receiving treatment within the trial, most because one centre allowed them to take questionnaires home for return by post. The other violation randomised the same child twice following a change of web servers at another site. Fortunately sensitivity analysis including these 29 children showed essentially the same results.[15] Figure 1b shows that, of the 308 fully compliant children, 256 (84%) returned questionnaires at 3 months, 266 (86%) did so at 6 months and imputation enabled us to analyse 293 (95%). Thus the incentive of shopping vouchers achieved much higher response rates than our targets. Hence the trial was better powered than planned.

Baseline characteristics

Table 1 shows characteristics of the 293 analysable participants at baseline.

Table 1: Characteristics of participants at baseline by allocated group

		EPIC kit	Treatment as usual
	**N (row %)**	190 (65%)	103 (35%)
DEMOGRAPHIC CHARACTERISTICS [n (%) unless specified]			
**Gender**	Male	85 (45)	49 (48)
	Female	105 (55)	54 (52)
**Age in years**	Range	6.3-18.9	6.4-18.4
	Mean (SD)	12.4 (3.0)	12.7 (3.2)
**Ethnicity**	White British	179 (94)	101 (98)
	Other	11 (6)	2 (2)
**Education & employment**	Secondary school	13 (42)	7 (33)
	Further Education College	13 (42)	12 (57)
	Other	5 (16)	1 (5)
	Age <16 so not asked	159	86
**Living situation**	Owner occupied house/flat	156 (82)	86 (83)
	Privately rented house/flat	20 (11)	6 (6)
	Housing assoc./local authority	14 (7)	11 (11)
**Years since diagnosis**	Range	0.8 – 16.7	1.2 – 15.7
	Mean (SD)	7.4 (3.8)	8.0 (3.9)
**Type of insulin administration**	Injections	167 (88)	87 (84)
	Pump	23 (12)	16 (16)
**Insulin regimen**	Once a day	2 (1)	-
	2 times a day	41 (25)	19 (22)

For peer review only - http://bmjopen.bmj.com/site/about/guidelines.xhtml

	3 times a day	14 (8)	10 (11)
	4 times a day	95 (57)	45 (52)
	Other (at least 5 times a day)	15 (9)	13 (15)
**Blood glucose tests**	None	1 (1)	1 (1)
	Once a day	4 (2)	1 (1)
	2 times a day	7 (4)	5 (5)
	3 times a day	31 (16)	16 (16)
	4 times a day	89 (47)	50 (49)
	Other (at least 5 times a day)	57 (30)	29 (28)
	Missing	1	1
**HbA_{1C} (%) (*mmol/mol*)** [1]	Range	5.9-14.0 *41.0-129.5*	6.0-13.7 *42.1-126.2*
	Mean	8.77/*72.3*	8.59/*70.4*

For peer review only

QOL BASELINE MEASURES AT TRIAL ENTRY			
Child self-report	PedsQL: Diabetes module (total scale score 100)	73.63 (14.68)	73.29 (12.17)
	PedsQL: Generic module (total scale score 100)	83.70 (12.36)	81.78 (12.63)
	EQ-5D utility score (total scale score 1)	0.9012 (0.1501)	0.8976 (0.1537)
	EQ-5D VAS (total scale score 100)	83.22 (16.98)	77.86 (18.89)
Parent proxy	PedsQL: Diabetes module (total scale score 100)	65.82 (15.35)	65.65 (14.02)
	PedsQL: Generic module (total scale score 100)	77.86 (14.66)	77.78 (14.43)
	EQ-5D utility score (total scale score 1)	0.8499 (0.1733)	0.8231 (0.1800)
	EQ-5D VAS (total scale score100)	83.02 (16.40)	79.96 (19.11)

[1] Pooled estimates.

For peer review only

Primary outcome

Tables 2 and 3 show that, of the five dimensions of the Diabetes PedsQL, only Worry showed adjusted scores significantly favouring self-management kits at three months: the mean child-reported difference was +5.87 with standard error [SE] of 2.19, generating statistical significance level (p) of 0.8% and a 95% confidence interval [CI] from +1.57 to +10.18. At six months, however, only Treatment Adherence achieved significance – in favour of treatment as usual: the mean child-reported difference was –4.68 with SE of 1.74, generating another p of 0.8% and 95% CI from –8.10 to –1.25. Even worse, intervention children reported significant adverse changes between three and six months on four of the five Diabetes PedsQL dimensions and thus on the total score: the mean child-reported difference was –3.20 with statistical significance level of 2.0% and 95% CI from –5.73 to –0.67.

Secondary outcomes

Moreover there was no evidence of change in HbA_{1c}. Participants started the trial with a mean baseline HbA_{1c} of 72 mmol/mol. Whether adjusted by baseline scores and stratification variables or not, this mean remained virtually unchanged during the follow up period: after three months self-managing children had reduced their adjusted HbA_{1c} by only –0.63 mmol/mol relative to comparator children (p=0.64; 95% CI from –3.31 to +2.04; after six months the reduction was only –0.19 mmol/mol (p=0.90; 95% CI from –3.07 to +2.70). At baseline only 16% of children across both treatment groups had achieved HbA_{1c} levels ≤ 58 mmol/mol, and at six months only 18%, thereby matching the national average in 2012/3.[5]

For peer review only - http://bmjopen.bmj.com/site/about/guidelines.xhtml

Tables 2 and 3 also show that the only statistically significant difference between groups in General PedsQL or EQ-5D-3L was that self-managing children reported that their School Functioning after six months was worse by –5.79 on average (p < 0.001; 95% CI from –9.21 to –2.36). Those intervention children also reported significant adverse changes between three and six months on two of the four Diabetes PedsQL dimensions and thus on the total score: the mean child-reported difference was –5.78 (p=0.002; 95% CI from –9.39 to –2.17).

However the parent-reported PedsQL scores, showed a different pattern from the child-reported scores: no delayed negative effects were apparent; instead comparator children were significantly better after three months in Diabetes Symptoms by –2.93 (p=0.036; 95% CI from –5.68 to –0.19); and in Treatment Adherence by –3.86 (p=0.026; 95% CI from –7.25 to –0.47).

Adverse events

Participating clinicians reported 31 serious adverse events to the trial team – 22 in the intervention group and 9 in the comparator group, yielding a relative risk of 1.33 (p=0.45; 95% CI from 0.63 to 2.77). The CPI, and the Chairs of the Data Monitoring & Ethics Committee and the Trial Steering Committee, reviewed and confirmed these SAEs. The CPI and research team judged that 4 SAEs from the Intervention Group and 2 from the Control Group were SARs possibly related to the EPIC intervention or to treatment as usual, yielding a relative risk of 1.08 (p=0.92; 95% CI from 0.20 to 5.82).

For peer review only - http://bmjopen.bmj.com/site/about/guidelines.xhtml

Table 2: Mixed models adjusted by stratification variables and baseline values BY treatment allocated

Outcome variable	Difference (Epic Pack– Treatment as usual)					Covariates/Cofactors sig at 5%		
	F(1,290)	p	Mean	s.e.	95% CI		F(1,290)	p
**Child self-report**								
**PedsQL: General**								
Total score	0.76	0.384	-0.96	1.10	-3.13 to 1.21	Baseline	275	<0.001
						Time-point	4.36	0.038
						Treatment group by time-point	5.13	0.024
Physical functioning	0.00	0.974	0.04	1.32	-2.56 to 2.65	Baseline	103	<0.001
						Time-point	5.38	0.021
						Treatment group by time-point	8.14	0.005
Emotional functioning	0.08	0.780	-0.51	1.83	-4.12 to 3.09	Baseline	184	<0.001
Social functioning	0.00	0.971	-0.05	1.46	-2.92 to 2.81	Baseline	181	<0.001
School functioning	4.26	0.040	-2.90	1.40	-5.66 to -0.14	Baseline	271	<0.001
						Treatment group by time-point	9.59	0.002
**PedsQL: Diabetes**								
Total score	0.07	0.798	-0.32	1.26	-2.80 to 2.16	Baseline	183	<0.001
						Treatment group by time-point	5.44	0.020
Diabetes symptoms	0.00	0.955	-0.09	1.55	-3.14 to 2.96	Baseline	170	<0.001
Treatment barriers	0.02	0.876	-0.27	1.71	-3.64 to 3.10	Baseline	174	<0.001
						Treatment group by time-point	4.19	0.042
Treatment adherence	2.60	0.108	-2.38	1.47	-5.28 to 0.52	Baseline	78	<0.001
						Treatment group-by-time-point	6.87	0.009
Worry	2.76	0.098	3.23	1.94	-0.60 to 7.05	Baseline	176	<0.001
						Treatment group by time-point	4.88	0.028
Communication	0.13	0.720	0.69	1.93	-3.10 to 4.48	Baseline	107	<0.001
						Treatment group-by-time-point	7.17	0.008
**EQ-5D**	0.00	0.960	.001	.018	-.034 to .036	Baseline	115	<0.001
						Gender	6.12	0.014

For peer review only - http://bmjopen.bmj.com/site/about/guidelines.xhtml

						Length of time since diagnosis	6.82	0.009
**EQ-5D: VAS**	0.42	0.520	1.10	1.70	-2.26 to 4.45	Baseline	125	<0.001
						Age	4.05	0.045
**Parent proxy**								
**PedsQL: General**								
Total score	0.66	0.417	0.94	1.15	-1.33 to 3.20	Baseline	264	<0.001
Physical functioning	2.61	0.107	2.22	1.38	-0.48 to 4.93	Baseline	94	<0.001
Emotional functioning	0.06	0.799	-0.48	1.86	-4.15 to 3.20	Baseline	143	<0.001
Social functioning	0.25	0.620	0.72	1.45	-2.13 to 3.57	Baseline	292	<0.001
						Age	5.28	0.022
School functioning	0.463	0.497	0.97	1.42	-1.84 to 3.77	Baseline	316	<0.001
						Time point	6.81	0.010
**PedsQL: Diabetes**								
Total score	2.36	0.125	-1.68	1.09	-3.82 to 0.47	Baseline	412	<0.001
Diabetes symptoms	1.44	0.232	-1.55	1.29	-4.09 to 1.00	Baseline	390	<0.001
Treatment barriers	0.57	0.452	-1.20	1.60	-4.36 to 1.95	Baseline	270	<0.001
						Age	5.67	0.018
Treatment adherence	6.15	0.014	-3.48	1.40	-6.23 to -0.72	Baseline	218	<0.001
Worry	0.15	0.697	-0.77	1.98	-4.66 to 3.12	Baseline	200	<0.001
						Time-point	9.49	0.002
Communication	0.11	0.742	0.67	2.04	-3.34 to 4.69	Baseline	171	<0.001
**EQ-5D**	1.82	0.178	-0.025	0.019	-0.062 to 0.012	Baseline	71	<0.001
**EQ-5D: VAS**	0.76	0.385	-1.34	1.54	-4.38 to 1.69	Baseline	171	<0.001
						Gender	5.22	0.023
**HbA1c**								
mmol/mol	0.11	0.740	-0.40	1.22	-2.80 to 1.99	Baseline	330	<0.001
	**F(1,290)**	**p**	**Mean**	**s.e.**	**95% CI**		**F(1,290)**	**p**

For peer review only - http://bmjopen.bmj.com/site/about/guidelines.xhtml

For peer review only

Table 3: Mixed models: mean effect at 3 & 6 months estimated from main model

Outcome variable	Time point	Difference (Epic Pack– Treatment as usual)					
		Mean	s.e.	95% CI	p	Change (6mth-3mth)	p
**Child self-report**							
**PedsQL: General**							
Total score	3mth	0.52	1.26	(-1.96 to 2.99)	0.682	-2.96	0.024
	6mth	-2.44	1.30	(-5.01 to 0.13)	0.063		
Physical functioning	3mth	2.16	1.59	(-0.96 to 5.29)	0.174	-4.24	0.005
	6mth	-2.08	1.44	(-4.92 to 0.76)	0.151		
Emotional functioning	3mth	-0.60	2.06	(-4.65 to 3.45)	0.769	0.18	0.932
	6mth	-0.42	2.19	(-4.73 to 3.89)	0.848		
Social functioning	3mth	0.55	1.62	(-2.64 to 3.75)	0.734	-1.21	0.455
	6mth	-0.66	1.72	(-4.03 to 2.72)	0.701		
School functioning	3mth	-0.01	1.63	(-3.21 to 3.20)	0.997	-5.78	0.002
	6mth	-5.79	1.74	(-9.21 to -2.36)	0.001		
**PedsQL: Diabetes**							
Total score	3mth	1.23	1.39	(-1.50 to 3.96)	0.375	-3.11	0.020
	6mth	-1.88	1.46	(-4.76 to 1.00)	0.200		
Diabetes symptoms	3mth	0.14	1.72	(-3.26 to 3.53)	0.938	-0.45	0.803
	6mth	-0.31	1.81	(-3.88 to 3.26)	0.865		
Treatment barriers	3mth	1.77	1.91	(-1.98 to 5.52)	0.355	-4.07	0.042
	6mth	-2.30	2.05	(-6.34 to 1.74)	0.263		
Treatment adherence	3mth	-0.08	1.69	(-3.40 to 3.25)	0.964	-4.76	0.009
	6mth	-4.68	1.74	(-8.10 to -1.25)	0.008		
Worry	3mth	5.87	2.19	(1.57 to 10.18)	0.008	-5.29	0.028
	6mth	0.58	2.37	(-4.09 to 5.25)	0.808		
Communication	3mth	3.66	2.10	(-0.47 to 7.80)	0.082	-5.94	0.008
	6mth	-2.28	2.34	(-6.89 to 2.32)	0.330		
**EQ-5D**	3mth	0.013	0.023	(-0.032 to 0.059)	0.568	-0.025	0.304
	6mth	-0.011	0.019	(-0.050 to 0.027)	0.555		
**EQ-5D: VAS**	3mth	0.58	1.83	(-3.02 to 4.18)	0.751	1.04	0.569
	6mth	1.62	2.03	(-2.37 to 5.60)	0.426		
**Parent proxy**							
**PedsQL: General**							
Total score	3mth	0.04	0.976	(-2.43 to 2.51)	0.976	1.79	0.114
	6mth	1.83	0.191	(-0.92 to 4.58)	0.191		
Physical functioning	3mth	1.53	1.53	(-1.49 to 4.55)	0.319	1.39	0.399
	6mth	2.92	1.66	(-0.36 to 6.19)	0.081		
Emotional functioning	3mth	-2.44	2.10	(-6.58 to 1.71)	0.248	3.93	0.068
	6mth	1.49	2.20	(-2.84 to 5.81)	0.499		
Social functioning	3mth	1.16	1.60	(-1.99 to 4.30)	0.470	-0.88	0.586
	6mth	0.28	1.71	(-3.09 to 3.65)	0.870		
School functioning	3mth	-0.53	1.72	(-3.91 to 2.85)	0.757	3.00	0.114

For peer review only - http://bmjopen.bmj.com/site/about/guidelines.xhtml

	6mth	2.47	1.70	(-0.88 to 5.82)	0.148		
**PedsQL: Diabetes**							
Total score	3mth	-2.29	1.20	(-4.64 to 0.07)	0.057	3.49	0.781
	6mth	1.20	1.38	(-3.78 to 1.65)	0.440		
Diabetes symptoms	3mth	-2.93	1.40	(-5.68 to -0.19)	0.036	2.77	0.074
	6mth	-0.16	1.61	(-3.33 to 3.01)	0.920		
Treatment barriers	3mth	-2.71	1.85	(-6.36 to 0.94)	0.145	3.01	0.145
	6mth	0.30	1.98	(-3.59 to 4.19)	0.880		
Treatment adherence	3mth	-3.86	1.72	(-7.25 to -0.47)	0.026	0.77	0.702
	6mth	-3.09	1.73	(-6.50 to 0.32)	0.076		
Worry	3mth	1.15	2.44	(-3.64 to 5.94)	0.637	3.84	0.161
	6mth	-2.69	2.38	(-7.37 to 1.98)	0.258		
Communication	3mth	1.04	2.43	(-3.74 to 5.83)	0.668	1.98	0.768
	6mth	3.02	2.36	(-4.34 to 4.94)	0.898		
**EQ-5D**	3mth	-0.024	0.023	(-0.069 to 0.020)	0.280	-0.001	0.963
	6mth	-0.026	0.022	(-0.070 to 0.019)	0.254		
**EQ-5D: VAS**	3mth	-2.00	1.78	(-5.50 to 1.51)	0.263	1.31	0.484
	6mth	-0.69	1.82	(-4.28 to 2.90)	0.706		
**HbA1c**							
mmol/mol	3mth	-0.63	1.36	(-3.31 to 2.04)	0.641	0.45	0.765
	6mth	-0.19	1.46	(-3.07 to 2.70)	0.899		

Sensitivity analyses

Our sensitivity analysis confirmed that participants with incomplete outcomes (withdrawals or those lost to follow up) did not change the findings of this trial.

Post-hoc analysis

Given the shortage of treatment effects and the varying size of the three age subgroups, we undertook post-hoc analysis to see if there was any evidence of an effect within individual age groups bands. We found no evidence of differences in outcomes by age group.

Cost consequences

We based economic analysis on 233 (80%) of the 293 children in the effectiveness analysis. We excluded 60 (20%) children with incomplete data on costs and service use because we could not be sure that these were missing at random. The mean total intervention unit cost of producing and administering intervention kits was £185 (Table 4). The mean total cost (NHS costs including intervention kit and administration costs) was £136 (bootstrapped 95% CI: -£52, £296) higher for the intervention group than for the comparator group, but this

For peer review only - http://bmjopen.bmj.com/site/about/guidelines.xhtml

difference was not statistically significant (Table 4A). We undertook bootstrapping with 1000 replicates to estimate a 95% confidence interval around this mean difference in costs and consequences between groups. For consequences, there were no significant mean differences for any outcome. Sensitivity analysis postulating that consultants instead of PDSNs see children in clinic but for the same 13.2 minutes, increased the mean difference in total costs of service use between groups from £136 to £182 (bootstrapped 95% confidence interval: -£9 to £339), but this difference was still not statistically significant.

For peer review only

For peer review only - http://bmjopen.bmj.com/site/about/guidelines.xhtml

Table 4A. Costs of producing and distributing self-management intervention kits and distributing them to participants by PDSNs in clinics in 2011 UK pounds (£)

Age group	6-10 years		11-15 years		16-18 years		Across all ages
Administration route	Injection	Pump	Injection	Pump	Injection	Pump	
Cost of self-management kit [a] (£)	11.57	16.67	12.09	17.19	22.07	28.29	
Cost of extra PDSN appointments [b] (£)	166.66	166.66	166.66	166.66	166.66	166.66	
**Total cost per participant (£)**	**178.23**	**183.33**	**178.75**	**183.85**	**188.73**	**194.95**	**184.64**
Number in Intervention Group	49	9	66	8	21	5	158
**Total cost of intervention (£)**	**8733**	**1650**	**11798**	**1471**	**3963**	**975**	**28590**
**Average cost per participant (£)**							**180.95**

Notes:

a. Including 'treatment cost' of producing and printing age-specific diabetes diaries, but not 'research cost' of developing these diaries.
b. Including 'treatment cost' of estimated time of Paediatric Diabetes Specialist Nurses (PDSNs) in teaching children about the kit, but not 'research cost' of research nurses distributing the kit.

For peer review only - http://bmjopen.bmj.com/site/about/guidelines.xhtml

Table 4B. Health service use costs and consequences by allocated group over 6 months

	Intervention kits (n=158) Mean (SD)	Treatment as usual (n=75) Mean (SD)	Intervention minus treatment as usual (bootstrapped 95% CIs)
Costs			
**Primary care (£)** [a]	67 (111)	61 (90)	6 (-22, 32)
**Secondary care (£)** [a]	454 (524)	504 (648)	-50 (-226, 103)
**EPIC intervention cost (£)** [a, b]	181 (4)	0 (0)	181
**Total cost / participant (£)**	702 (558)	566 (664)	136 (-52, 296)
Consequences			
Participant self-report:			
**QALYs**	0.446 (0.0741)	0.447 (0.0784)	-0.001 (-0.0209, 0.0189)
Parent-proxy:			
**QALYs**	0.415 (0.0785)	0.418 (0.0831)	-0.003 (-0.0238, 0.0188)

Notes:

[a] Mean (SD) total cost per participant (£)

[b] Cost of intervention includes: the 'treatment costs' of producing and distributing the kit to participants by Paediatric Diabetes Specialist Nurses in clinics; those of producing and distributing the intervention diabetes diary; but not the 'research costs' of developing kit or diary.

For peer review only - http://bmjopen.bmj.com/site/about/guidelines.xhtml

Process Evaluation

We present key findings from the process evaluation [12] to illustrate the wider context within which children were initially excited but thereafter did not engage as intended with diabetes self-management generally or use the standardised kits specifically. We offer an explanation as to how the context was created for this mechanism to occur.

Normalisation in children's self-management and relationships with professionals

After initial excitement, most children said that they did not use the EPIC kits as intended; a few rejected them completely and put them out of sight, for example in the loft. Analysis of the words, messages and images in children's self-management information found that they generally presented rules to manage diabetes supported by images of being 'normal' like other children if they followed those rules and did what professionals said. The process evaluation showed that these authoritarian normalisation messages did not always resonate with children, especially teenagers, as they did not feel normal because their life was frequently defined by diabetes, which they disliked. The presence of the EPIC kits and the messages they contained caused increasing levels of worry and anxiety. So they tried to hide diabetes by not making 'self-management' visible to themselves or others. Hence few children took a diabetes diary to school or wanted to test their blood sugar levels.

Children's inability to associate blood glucose tests with better management

Baseline questionnaires from 308 children and their parents at entry to the trial show that they knew how many times a day they should record their blood glucose. In reality most children, especially teenagers and irrespective of allocation, did not use or even see the need to record or observe trends in blood glucose levels to titrate their insulin dose. Of those interviewed in the intervention treatment group, around half of 6-10 year olds, less than half of 11-15 year olds, but only around 20% of 16-18 year olds said that they or their parents recorded blood glucose levels; fewer still appeared to use levels to titrate insulin doses. Children more commonly neglected the age-appropriate self-management information provided. Many children thought that they were recording this information for the benefit of diabetes professionals and made no link between blood glucose testing and gaining better diabetes control.

For peer review only - http://bmjopen.bmj.com/site/about/guidelines.xhtml

Children's ignorance of risky behaviour and long-term complications of diabetes

By not titrating insulin doses to blood glucose levels, many children took risks with their diabetes-related health; many teenagers appeared unconcerned about the potential consequences. Some parents said they wanted to protect their children from receiving information on risks and complications, whereas others wanted to expose their children to the reality of life threatening complications like renal failure. Discourse analysis showed that: children's diabetes information resources for ages 6-10 years rarely mentioned risks or complications of poorly controlled diabetes; while those for ages 11-15 years were usually vague about serious risks and long-term complications of poorly controlled diabetes. In contrast information distributed on entry to adult diabetes services was explicit about risks of long-term complications and the resulting need for self-management to minimise these.

Promotion of intervention by diabetes professionals and parents

Most diabetes teams did not actively engage with the intervention kits or encourage their use in routine consultations. Around a third of children approached declined to participate. Often there were modifications to intended intervention delivery; for example the research nurse, not a member of the child's diabetes team, gave the kit and diary to the child. There was little individualisation or tailoring of intervention kits. Around half the PDSNs regulated the information given to children and knowingly withheld or removed information on lifestyle issues and risks of complications before distribution to children. From the kit for ages 11-15 distributed to 103 participants, professionals reported removing several topics as inappropriate: sex and beyond (12 times), drinking alcohol (11 times), body piercing (8 times), and carbohydrate awareness (4 times). After children had received the kits, many parents had removed anything they thought unsuitable, notably the 'lifestyle' resources, if not already removed by the PDSN.

For peer review only

Discussion

Principal findings

This pragmatic trial found no evidence of benefit from age-appropriate diabetes self-management kits for children. Of the five dimensions of the Diabetes PedsQL, Worry showed adjusted scores significantly favouring self-management kits at three months but Treatment Adherence significantly favoured controls at six months. Furthermore children using self-management kits reported significantly worse changes between three and six months on four of the five Diabetes PedsQL dimensions and thus on the total score. There was no evidence of change in HbA1c; only 18% of participants in each group achieved recommended levels at 6 months. This was apparently because the kits alienated children and parents, and their use of kits and recording of blood glucose was poor. Moreover five [29-33] other contemporaneous UK trials of educational interventions for childhood diabetes reported no benefit and little rapport between children, their parents, clinic staff, and the interventions. The other five interventions comprised structured diabetes education, family-based diabetes education and support [30-33] and training in communication skills[29]. None of these six UK trials (including EPIC) showed any difference in HbA_{1c} between groups; or evidence of any other benefit. EPIC is the first trial to make clear a deleterious intervention effect over time. As fewer than 20% of all 2018 children across the six trials achieved glycaemic control (pre 2015 HbA1c target of ≤58 mmol/mol), this meant that over 80% of children were at risk of serious complications. All six trials reported concerns about intervention fidelity. Attendance at additional diabetes teaching sessions was highly variable, and those with the highest HbA_{1c} were least likely to attend. A recent systematic review of ten trials of education and psychoeducation interventions (Including the five trials above but excluding EPIC as HbA1c had not been fully analysed at the time) showed a non-significant reduction in HbA1c attributable to the intervention (pooled SMD = -0.06, 95% CI: -0.21 to 0.09).[34]

Our parallel analysis of the words and images in diabetes resources provides new insights into the potential source of the surprisingly adverse effects of the kits. These stem in part from children's rejection of 'unwelcome' information which labelled them as different in having diabetes and authoritarian instructions like 'take me with you wherever you go'.

For peer review only - http://bmjopen.bmj.com/site/about/guidelines.xhtml

More generally the discourse of 'normalisation' through optimal management and insulin as a social enabler appears counterproductive in promoting desired behaviour change. Poor relationships between children, parents and diabetes professionals is another reason for lack of 'compliance' with diabetes professionals' expectations. In short diabetes communicators do not yet know how to convey effective messages to children. One trial (DEPICTED)[29], which attempted to train health-care professionals do this, also failed to show any benefit.

Strengths and weaknesses of this study

We based the EPIC kit on extensive research with children, parents and professionals; and met international standards for patient information.[12-13] We powered the EPIC trial to detect plausible effect sizes for the entire age-range, and surpassed recruitment targets.[20,21] Particular strengths included the preparatory discourse analysis of children's diabetes self-management information and the process evaluation in which children assessed the ways in which they received key diabetes self-management information. In retrospect the major weakness was the unpopularity of the EPIC kit.

Interpretation

Lack of progress in meeting the NICE target for HbA_{1c} in children stimulated the commissioning of this and five other contemporaneous trials by UK funders to test various approaches to promote optimal self-management. Other promising developments since the delivery of this trial, include electronic management systems, diabetes phone and iPad applications, and personalised web-based diabetes training programmes.[27-30] However these are unlikely to benefit children if, despite receiving good information, they still do not make the link between blood glucose testing and achieving glycaemic control through insulin, diet and lifestyle management.

In contrast, international studies show that children can achieve acceptable glycaemic control through investment in structured education and patient management following diagnosis, with outcomes ranging from 29% to over 50% of children achieving an HbA1c level less than ≤58mmol/mol [36-36] this is much higher than the 18% achieved by EPIC & the UK in general. The Hvidøre Study Group [35] identified management from the diagnosis of the

For peer review only - http://bmjopen.bmj.com/site/about/guidelines.xhtml

disease, positive and shared attitudes within diabetes teams and greater patient empowerment as factors that enhanced glycaemic control. Though we understand these factors, we do not know why UK children cannot achieve similar standards.

Implications for practice and research

Our process evaluation and discourse analysis identified two problems which have not yet been adequately addressed. First, we need to understand better how children respond to 'authority' in relationships with diabetes professionals. Expected regular attendance at outpatient clinics, the normative nature of most self-management information, and the withholding of requested lifestyle information all reinforce the power imbalance. Despite the intended focus on 'normalisation', children feel different, dislike being different, and tend to reject any intervention that reminds them of that difference. The second problem is that children, parents and professionals cannot reconcile clinical risk definitions and personal ones. Children assess risks differently from diabetes professionals and take rational decisions about what is acceptable for them. To overcome this, professionals may need to address risk collaboratively rather than hierarchically and this would require a complete rethink as to how diabetes services are designed and delivered. Motivational interviewing has, for example, been shown to be an effective method of facilitating behavioural changes in teenagers with type 1 diabetes with subsequent improvement in their glycaemic control.[37]

There are also implications for trial methods and conduct. First, we need to encourage research and clinical teams to implement complex interventions like this rigorously but also flexibly. Secondly, as the care of children's diabetes varies widely across the UK, there is merit in engaging the diabetes teams likely to contribute to a planned trial in design and planning.

For peer review only - http://bmjopen.bmj.com/site/about/guidelines.xhtml

References

1. NHS Digital. National diabetes audit, 2015-16 report 2a: Complications and mortality (complications of diabetes) England and Wales 2017:https://files.digital.nhs.uk/pdf/4/t/national_diabetes_audit__2015-16__report_2a.pdf.

2. International Society for Pediatric and Adolescent Diabetes. ISPAD clinical practice consensus guidelines 2009. www.ispad.org/resource-type/ispad-clinical-practice-consensus-guidelines- 2009. Accessed 10.3, 2013.

3. National Institute for Health and Care Excellence. Type 1 diabetes: Diagnosis and management of type 1 diabetes in children and young people. *London: National Institute for Clinical Excellence*. 2004.

4. Royal College of Paediatrics and Child Health (RCPCH). Growing up with diabetes: Children and Young people with diabetes in ENGLAND. . *RCPCH, London.* 2009.

5. Scottish Intercollegiate Guidelines Network. Management of diabetes: A national clinical guideline. 2010;116.

6. NHS Information Centre. National diabetes paediatric audit report 2009–10. *Leeds: NHS Information Centre for Health and Social Care; URL: www.diabetes.org.uk/Documents/ News/Paediatric_Diabetes_Audit130711.pdf*. 2011.

7. NHS Information Centre. National diabetes paediatric audit report 2011–12. *Leeds: NHS Information Centre for Health and Social Care http://www rcpch ac uk/child-health/standards-care/clinical-audit-and-quality-improvement/national-paediatric-diabetes-au-1*. 2012.

8. NHS Information Centre. National paediatric diabetes audit 2012-13. . 2013;http://www.rcpch.ac.uk/system/files/protected/page/NPDA%202012-13%20Core%20Report%202nd%20FINAL%20v%203.3.pdf.

9. NICE National Institute for Health and care Excellence. Diabetes (type 1 and type 2) in children and young people: Diagnosis and management. . 2015;https://www.nice.org.uk/guidance/ng18/resources/diabetes-type-1-and-type-2-in-children-and-young-people-diagnosis-and-management-pdf-1837278149317.

10. Edwards D, Noyes J, Lowes L, Haf Spencer L, Gregory JW. An ongoing struggle: A mixed-method systematic review of interventions, barriers and facilitators to achieving optimal self-care by children and young people with type 1 diabetes in educational settings. *BMC Pediatr*. 2014;14:228-doi: 10.1186/1471-2431-14-228.

11. Allen D, Channon S, Cohen D, Hooten N, Lowes L, Owens D, et al. The transition from paediatric to adult diabetes services: What works, for whom and in what circumstances? final report. *NIHR Service Delivery and Organisation Programme*. 2010.

12. Noyes JP, Lowes L, Whitaker R, Allen D, Carter C, Edwards RT, et al. Developing and evaluating a child-centred intervention for diabetes medicine management using mixed methods and a multicentre randomised controlled trial. *Health Serv Deliv Res*. 2014;2(8):1-482.

13. Williams A, Noyes J, Chandler-Oatts J, Allen D, Brocklehurst P, Carter C, et al. Children's health information matters: Researching the practice of and requirements for age appropriate health information for children and young people. final report. *NIHR Service Delivery and Organisation Programme.* 2011.

For peer review only - http://bmjopen.bmj.com/site/about/guidelines.xhtml

14. Noyes J, Williams A, Allan D, Brocklehurst P, Carter C, Gregory J, et al. Evidence into practice: Evaluating a child-centred intervention for diabetes medicine management: The EPIC project. *BMC Pediatrics*. 2010;10(70):3-12.

15. Russell D, Hoare Z, Whittaker R, Whittaker C, Russell I. Generalized method for adaptive randomization in clinical trials. *Stat Med*. 2010;30:922-924.

16. Varni J, Burwinkle T, Jacobs J, Gottschalk M, Kaufman F, Jones K. The PedsQLTM in type 1 and type 2 diabetes. reliability and validity of the pediatric quality of life InventoryTM generic core scales and type 1 diabetes module. *Diabetes Care*. 2003;26:631-637.

17. Varni JW, Burwinkle TM, Seid M. The PedsQL™ as a pediatric patient-reported outcome: Reliability and validity of the PedsQL™ measurement model in 25,000 children. . *Expert Rev Pharmacoecon Outcomes Res*. 2005;5:705-719.

18. Euroqual. Eq-5d. http://www.euroqol.org/2018.

19. Beecham J, & Knapp M. Costing psychiatric interventions. In: Thornicroft G, Brewin C, Wing J., ed. *Measuring mental health needs.* Oxford: Oxford University Press; 1992:200-224.

20. Murphy H, Rayman G, Skinner T. Psycho-educational interventions for children and young people with type 1 diabetes. *Diabet Med*. 2006;23:935-943.

21. Hampson S, Skinner T, Hart J, Storey L, Gage H, Foxcroft D, et al. Effects of educational and psychosocial interventions for adolescents with diabetes mellitus: A systematic review . *Health Technol Assess*. 2001;5(10)(10).

22. Van Buuren S, Brand JPL, Groothuis-Oudshoorn K, Rubin D. Fully conditional specification in multivariate imputation. *Journal of Statistical Computation and Simulation*. 2006;76(12):1049-1064.

23. Khutoryansky NM HW. Imputation techniques using SAS software for incomplete data in diabetes clinical trials. 2001:335-359.

24. Curtis L. Unit costs of health and social care 2011. https://www.pssru.ac.uk/pub/uc/uc2011/uc2011.pdf

25. Department of Health. 2010-11 reference costs publication. 2011. https://assets.publishing.service.gov.uk/government/uploads/system/uploads/attachment_data/file/216345/dh_122794.pdf

26. Glick H Doshi J, Sonnad S, Polsky D. *Economic evaluation in clinical trials. .* Oxford: Oxford University Press; 2007.

27. Ritchie J. & Spencer L. Qualitative data analysis for applied policy research. In: Bryman A. & Burges R, ed. *Analysing qualitative data.* ; 1994:173–194.

28. Silverman D. *Interpreting qualitative data. methods for analysing talk, text and interaction.* 2nd edn ed. London: Sage Publications; 2001.

29. Robling M, McNamara R, Bennert K, Butler C, Channon S, Cohen D, et al. Training health care practitioners in the 'Talking diabetes' consulting skills intervention: A cluster randomized controlled trial of effects on blood glucose control and quality of life in children with type 1 diabetes (the DEPICTED study). *British Medical Journal*. 2012;26(e2359.):1-17.

For peer review only - http://bmjopen.bmj.com/site/about/guidelines.xhtml

30. Price KJ, Wales J, Eiser C, Knowles J, Heller S, Freeman J, Brennan A, McPherson A, Wellington J. Does an intensive self-management structured education course improve outcomes for children and young people with type 1 diabetes? the kids in control OF food (KICk-OFF) cluster-randomised controlled trial protocol. *BMJ Open*. 2013;3(1):e002429.

31. Murphy H, Wadham C, Hassler-Hurst et al, on behalf of Families and Adolescents Communication and Teamwork Study (FACTS) Group. Randomized trial of a diabetes self-management education and family teamwork intervention in adolescents with type 1 diabetes. *Diabetic Medicine*. 2012;29:249-54.

32. Christie D, Thompson R, Sawtell M, Allen E, Cairns J, Smith F, Jamieson E, Hargreaves K, Ingold A, Brooks L, Wiggins M, Oliver S, Jones R,Elbourne D, Santos A, Wong IC, O'Neill S, Strange V, Hindmarsh P, Annan F, Viner R. Structured, intensive education maximising engagement, motivation and long-term change for children and young people with diabetes: A cluster randomised controlled trial with integral process and economic evaluation - the CASCADE study. . *Health Technol Assess*. 2014;18:1-202.

33. Coates V, Chaney D, Bunting B, Shorter GW, Shevlin M, et al. (2013) Evaluation of the Effectiveness of a Structured Diabetes Education Programme (CHOICE) on Clinical Outcomes for Adolescents with Type 1 Diabetes: A Randomised Controlled Trial. J Diabetes Metab 4:280. doi: 10.4172/2155-6156.1000280

34. Charalampopoulos D, Hesketh KR, Amin R, Paes VM, Viner RM, Stephenson T Psycho-educational interventions for children and young people with Type 1 Diabetes in the UK: How effective are they? A systematic review and meta-analysis. PLoS ONE 2017 12(6): e0179685. https://doi.org/10.1371/journal.pone.0179685

35. Lang K, Sassman H, von Schütz W, Kordonouri O, Danne. Prerequisites for age-appropriate education in type 1 diabetes: A model programme for paediatric diabetes education in Germany. *Pediatr Diabetes*. 2007;6:63-74.

36. Mortensen H HP. Comparison of metabolic control in a cross-sectional study of 2,873 children and adolescents with IDDM from 18 countries. the hvidøre study group on childhood diabetes. *Diabetes Care*. 1997;20:714-720.

37. Channon SJ, Huws-Thomas MV, Rollnick S, Hood K, Cannings-John RL, Rogers C, Gregory JW. A Multicenter Randomized Controlled Trial of Motivational Interviewing in Teenagers With Diabetes. Diabetes Care. 2007 Jun;30(6):1390-5)

Competing Interest Statement

Professor John Gregory has received payments from Pfizer, Bayer and Ipsen for lectures, development of educational presentations and travel/accommodation to attend scientific meetings and advisory board meetings. His employer (Cardiff University) has also received funding from Novo Nordisk to support the development of patient-support materials used in the Development and Evaluation of a Psychosocial Intervention for Children and Teenagers Experiencing Diabetes (DEPICTED) research study. The remaining authors declare no competing interests

For peer review only

Author contributions

JN and ITR designed the original trial. DE, LS and STY contributed to data collection and analysis. RTE (health economist) and RW, YS (trial statisticians). CC undertook the discourse analysis. ITR and DR undertook the trial reanalysis with JN and JWG. All authors agreed the final manuscript.

Acknowledgements

Emerita Professors Anne Williams and Lesley Lowes. Jan Sharp and Professor Jo Rycroft-Malone. Professor Tim Barrett was independent Chair of the Trial Steering Group. Dr Chris Foy is independent Chair of the Data Monitoring and Ethics Committee (DMEC). Rachel Harris, diabetes nurse specialist was independent member of DMEC. Yvonne Rees-Coleman and Nina Phillips, were independent lay members of the Trial Steering Group. We thank the children, parents and staff who participated in the trial and process evaluation.

Data sharing statement

Trial data not reported in this paper are not available for sharing.

Funding

National Institute for Health Research

Grants: SDO/08/1718/145 and SDO/211/2007.

For peer review only

Figure 1a: CONSORT diagram from screening to analysis.

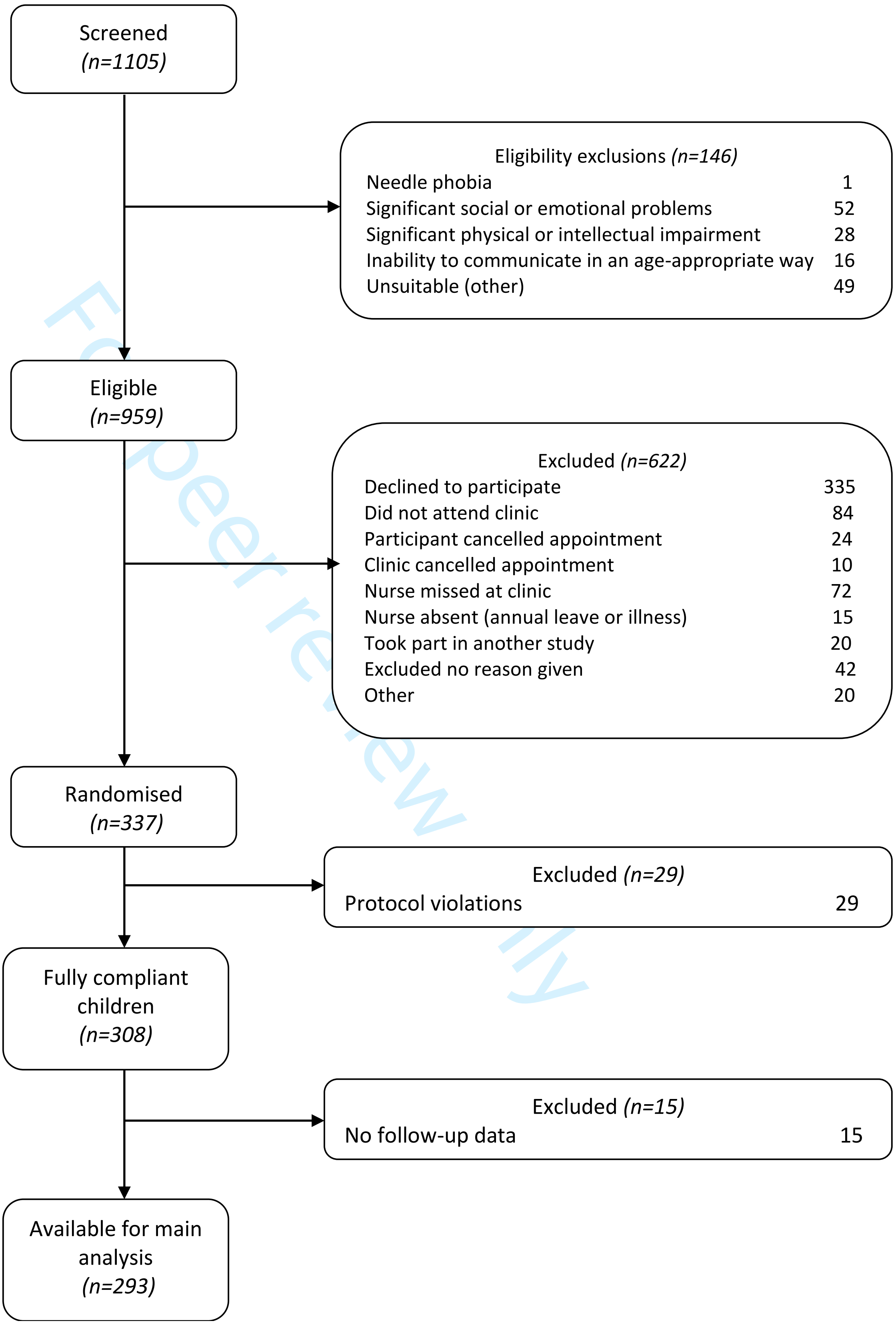

For peer review only

Figure 1b: CONSORT diagram, showing missing data from screening to analysis

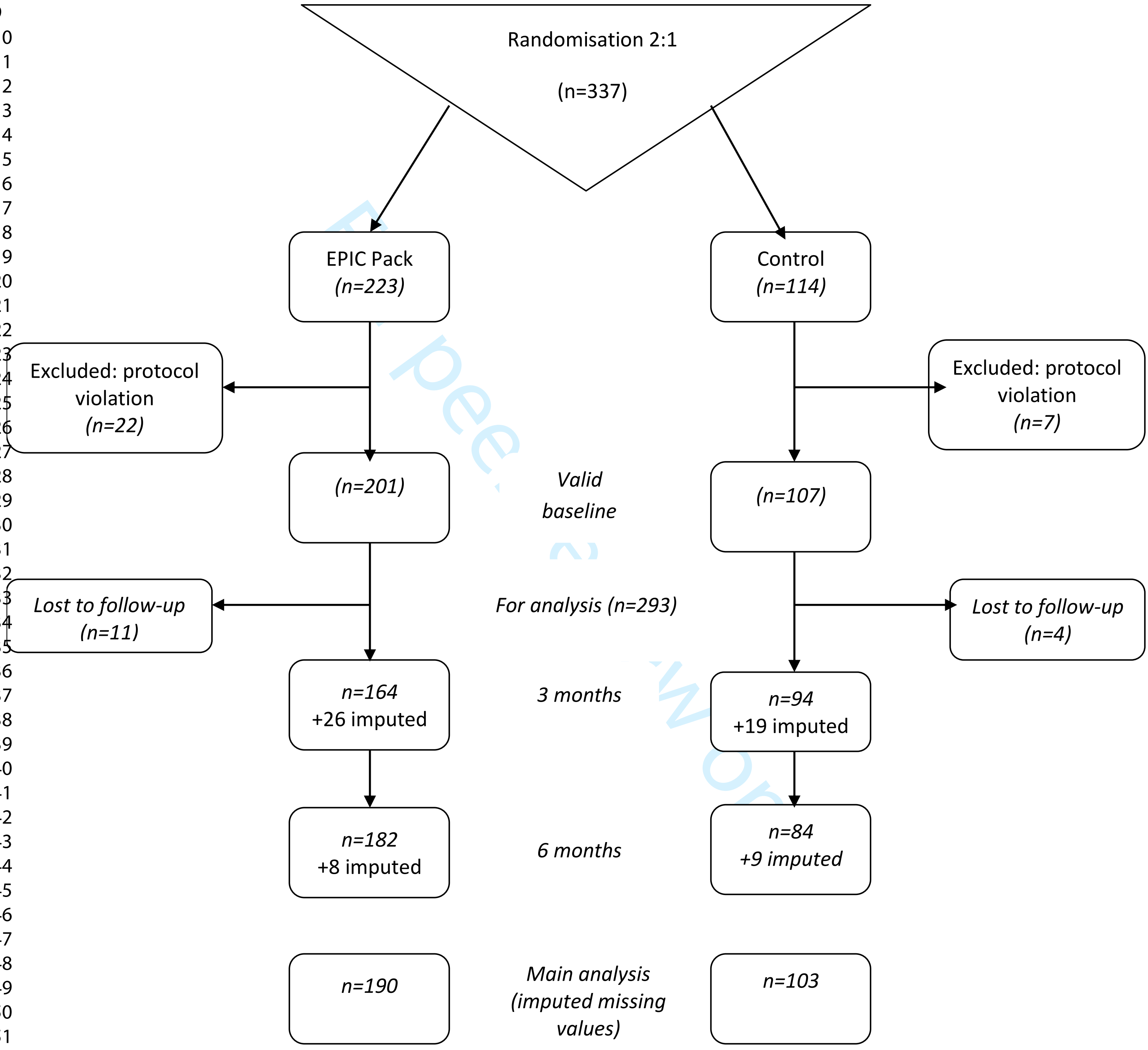

CONSORT 2010 checklist of information to include when reporting a randomised trial*

Section/Topic	Item No	Checklist item	Reported on page No
**Title and abstract**			
	1a	Identification as a randomised trial in the title	No as not just reporting a RCT
	1b	Structured summary of trial design, methods, results, and conclusions (for specific guidance see CONSORT for abstracts)	2-3
**Introduction**			
Background and objectives	2a	Scientific background and explanation of rationale	5-6
	2b	Specific objectives or hypotheses	5
**Methods**			
Trial design	3a	Description of trial design (such as parallel, factorial) including allocation ratio	6-10
	3b	Important changes to methods after trial commencement (such as eligibility criteria), with reasons	
Participants	4a	Eligibility criteria for participants	7
	4b	Settings and locations where the data were collected	7
Interventions	5	The interventions for each group with sufficient details to allow replication, including how and when they were actually administered	6-7
Outcomes	6a	Completely defined pre-specified primary and secondary outcome measures, including how and when they were assessed	8
	6b	Any changes to trial outcomes after the trial commenced, with reasons	
Sample size	7a	How sample size was determined	9
	7b	When applicable, explanation of any interim analyses and stopping guidelines	
Randomisation:			
Sequence generation	8a	Method used to generate the random allocation sequence	7
	8b	Type of randomisation; details of any restriction (such as blocking and block size)	7
Allocation concealment mechanism	9	Mechanism used to implement the random allocation sequence (such as sequentially numbered containers), describing any steps taken to conceal the sequence until interventions were assigned	8
Implementation	10	Who generated the random allocation sequence, who enrolled participants, and who assigned participants to	7

For peer review only - http://bmjopen.bmj.com/site/about/guidelines.xhtml
For peer review only

		interventions	
Blinding	11a	If done, who was blinded after assignment to interventions (for example, participants, care providers, those assessing outcomes) and how	8
	11b	If relevant, description of the similarity of interventions	
Statistical methods	12a	Statistical methods used to compare groups for primary and secondary outcomes	9
	12b	Methods for additional analyses, such as subgroup analyses and adjusted analyses	9
**Results**			
Participant flow (a diagram is strongly recommended)	13a	For each group, the numbers of participants who were randomly assigned, received intended treatment, and were analysed for the primary outcome	11
	13b	For each group, losses and exclusions after randomisation, together with reasons	11 and figures
Recruitment	14a	Dates defining the periods of recruitment and follow-up	2
	14b	Why the trial ended or was stopped	
Baseline data	15	A table showing baseline demographic and clinical characteristics for each group	12-13
Numbers analysed	16	For each group, number of participants (denominator) included in each analysis and whether the analysis was by original assigned groups	Figure 1
Outcomes and estimation	17a	For each primary and secondary outcome, results for each group, and the estimated effect size and its precision (such as 95% confidence interval)	15-18
	17b	For binary outcomes, presentation of both absolute and relative effect sizes is recommended	
Ancillary analyses	18	Results of any other analyses performed, including subgroup analyses and adjusted analyses, distinguishing pre-specified from exploratory	18
Harms	19	All important harms or unintended effects in each group (for specific guidance see CONSORT for harms)	14
**Discussion**			
Limitations	20	Trial limitations, addressing sources of potential bias, imprecision, and, if relevant, multiplicity of analyses	25
Generalisability	21	Generalisability (external validity, applicability) of the trial findings	25-6
Interpretation	22	Interpretation consistent with results, balancing benefits and harms, and considering other relevant evidence	25-6
**Other information**			
Registration	23	Registration number and name of trial registry	2
Protocol	24	Where the full trial protocol can be accessed, if available	6
Funding	25	Sources of funding and other support (such as supply of drugs), role of funders	30

For peer review only

*We strongly recommend reading this statement in conjunction with the CONSORT 2010 Explanation and Elaboration for important clarifications on all the items. If relevant, we also recommend reading CONSORT extensions for cluster randomised trials, non-inferiority and equivalence trials, non-pharmacological treatments, herbal interventions, and pragmatic trials. Additional extensions are forthcoming: for those and for up to date references relevant to this checklist, see www.consort-statement.org.

For peer review only

For peer review only - http://bmjopen.bmj.com/site/about/guidelines.xhtml

BMJ Open

Standardised self-management kits for children with type 1 diabetes: pragmatic randomised trial of effectiveness and cost-effectiveness

Journal:	*BMJ Open*
Manuscript ID	bmjopen-2019-032163.R1
Article Type:	Original research
Date Submitted by the Author:	20-Dec-2019

Complete List of Authors:	Noyes, Jane; Bangor University, School of Social Sciences<br>Allen, Davina; Cardiff University<br>Carter, Cynthia; Cardiff University, School of Journalism, Media and Culture<br>Edwards, Deborah; Cardiff University, School of Healthcare Sciences<br>Edwards, Rhiannon; Bangor University, Centre for Health Economics & Medicines Evaluation<br>Russell, Daphne; Swansea University, College of Medicine<br>Russell, Ian; Swansea University, Medicine<br>Spencer, Llinos; Bangor University, Centre for Health Economics and Medicine Evaluation<br>Sylvestre, Yvonne; University of Manchester , Manchester Clinical Trials Unit<br>Whitaker, Rhiannon; Whitaker Research Ltd. ,<br>Yeo, S. T.; University of Bangor School of Healthcare Sciences, Centre for Health Economics and Medicines Evaluation (CHEME)<br>Gregory, John; Cardiff University, School of Medicine

**Primary Subject Heading**:	Paediatrics
Secondary Subject Heading:	Health services research, Diabetes and endocrinology
Keywords:	Paediatric endocrinology < DIABETES & ENDOCRINOLOGY, HEALTH SERVICES ADMINISTRATION & MANAGEMENT, Health economics < HEALTH SERVICES ADMINISTRATION & MANAGEMENT

SCHOLARONE™
Manuscripts

I, the Submitting Author has the right to grant and does grant on behalf of all authors of the Work (as defined in the below author licence), an exclusive licence and/or a non-exclusive licence for contributions from authors who are: i) UK Crown employees; ii) where BMJ has agreed a CC-BY licence shall apply, and/or iii) in accordance with the terms applicable for US Federal Government officers or employees acting as part of their official duties; on a worldwide, perpetual, irrevocable, royalty-free basis to BMJ Publishing Group Ltd ("BMJ") its licensees and where the relevant Journal is co-owned by BMJ to the co-owners of the Journal, to publish the Work in this journal and any other BMJ products and to exploit all rights, as set out in our licence.

The Submitting Author accepts and understands that any supply made under these terms is made by BMJ to the Submitting Author unless you are acting as an employee on behalf of your employer or a postgraduate student of an affiliated institution which is paying any applicable article publishing charge ("APC") for Open Access articles. Where the Submitting Author wishes to make the Work available on an Open Access basis (and intends to pay the relevant APC), the terms of reuse of such Open Access shall be governed by a Creative Commons licence – details of these licences and which Creative Commons licence will apply to this Work are set out in our licence referred to above.

Other than as permitted in any relevant BMJ Author's Self Archiving Policies, I confirm this Work has not been accepted for publication elsewhere, is not being considered for publication elsewhere and does not duplicate material already published. I confirm all authors consent to publication of this Work and authorise the granting of this licence.

For peer review only

TITLE PAGE

Standardised self-management kits for children with type 1 diabetes: pragmatic randomised trial of effectiveness and cost-effectiveness

Corresponding Author: Jane Noyes,
Address: Neuadd Ogwen, School of Social Sciences, Bangor University, Wales, LL57 2DG, UK
Email: jane.noyes@bangor.ac.uk
Phone:+44 (0) 1248 388519
Twitter: @Jane_Noyes
Orchid: https://orcid.org/0000-0003-4238-5984

Co-authors:
Davina Allen, Professor of Nursing, School of Healthcare Sciences, Cardiff University, Eastgate House, Newport Road, Cardiff, United Kingdom, CF24 0AB, Email: allenda@cardiff.ac.uk

Cynthia Carter, Reader, School of Journalism, Media and Culture Cardiff University, Two Central Square, Cardiff, Wales, UK CF10 1FS email:cartercl@cardiff.ac.uk Orcid: https://orcid.org/0000-0002-5424-9835

Deborah Edwards, Research Associate, School of Healthcare Sciences, Cardiff University, Eastgate House, 35-43 Newport Road / Cardiff / CF24 0AB
Email: edwardsdj@cardiff.ac.uk. https://orcid.org/0000-0003-1885-9297

Rhiannon T Edwards, Co- Director Centre for Health Economics and Medicines Evaluation, Ardudwy Hall, Bangor University, Bangor, Gwynedd, LL57 2PZ, Email: r.t.edwards@bangor.ac.uk

Daphne Russell, Senior Trial Statistician, Swansea University Medical School, Singleton Park Swansea, SA2 8PP. E-mail: daphne.russell@cantab.net

Ian T Russell, Emeritus Professor of Clinical Trials, Swansea University Medical School, Singleton Park, Swansea, SA2 8PP i.t.russell@swansea.ac.uk

Llinos H Spencer, Research Officer, Centre for Health Economics and Medicines Evaluation, Bangor University, Neuadd Ardudwy, Normal Site, Bangor, Gwynedd, LL57 2PZ
Email: l.spencer@bangor.ac.uk. Orcid: https://orcid.org/0000-0002-7075-8015

Yvonne Sylvestre, Statistician, Christie Hospital NHS Trust, Manchester, UK. Email: ysylvestre@gmail.com

Rhiannon Whitaker, Statistician, Whitaker Research Ltd, 8 Whitehall Road, Rhos on Sea, LL28 4HW. Email: rhiannon@whitres.co.uk

Seow Tien Yeo, Research Officer (Health Economics), Centre for Health Economics and Medicines Evaluation (CHEME), School of Health Sciences, Bangor University, Ardudwy Building, Normal Site, Bangor, Gwynedd, LL57 2PZ. Email: s.t.yeo@bangor.ac.uk Orcid: http://orcid.org/0000-0001-7496-1742

John W Gregory, Professor in Paediatric Endocrinology & Honorary Consultant, Division of Population Medicine, School of Medicine, Cardiff University, Heath Park, Cardiff, CF14 4XN.
Email: wchjwg@Cardiff.ac.uk

For peer review only - http://bmjopen.bmj.com/site/about/guidelines.xhtml

Standardised self-management kits for children with type 1 diabetes: pragmatic randomised trial of effectiveness and cost-effectiveness

Abstract

Objective: To estimate the effectiveness of standardised self-management kits for children with type 1 diabetes.

Design: Pragmatic trial with randomisation ratio of 2 intervention: 1 control. Qualitative process evaluation.

Setting: 11 diabetes clinics in England and Wales.

Participants: Between February 2010 and August 2011 we validly randomised 308 children aged six to 18 years; 201 received the intervention.

Intervention: We designed kits to empower children to achieve glycaemic control, notably by recording blood glucose and titrating insulin. The comparator was usual treatment.

Outcome measures at 3 and 6 months: Primary: Diabetes PedsQL. Secondary: HbA1c; General PedsQL; EQ-5D; healthcare resource use.

Results: Of the five Diabetes PedsQL dimensions, Worry showed adjusted scores significantly favouring self-management kits at three months (mean child-reported difference = +5.87; standard error [SE] = 2.19; 95% confidence interval [CI] from +1.57 to +10.18; p = 0.008); but Treatment Adherence significantly favoured controls at six months (mean child-reported difference = -4.68; SE = 1.74; 95% CI from -8.10 to -1.25; p = 0.008). Intervention children reported significantly worse changes between three and six months on four of the five Diabetes PedsQL dimensions and on the total score (mean difference = -3.20; SE = 1.33; 95% CI from -5.73 to -0.67; p = 0.020). There was no evidence of change in HbA1c; only 18% of participants in each group achieved recommended levels at 6 months. No serious adverse reactions attributable to the intervention or its absence were reported.

Use of kits was poor. Few children or parents associated blood glucose readings with better glycaemic control. The kits, costing £185, alienated many children and parents.

Conclusions: Standardised kits showed no evidence of benefit, inhibited diabetes self-management and increased worry. Future research should study relationships between children and professionals, and seek new methods of helping children and parents to manage diabetes.

For peer review only - http://bmjopen.bmj.com/site/about/guidelines.xhtml

Trial registration: ISRCTN17551624

Funding: National Institute for Health Research

Kudos plain language summary

Children and their parents find it very challenging to manage their type 1 diabetes well. Daily self-management is complex and involves adjusting the amount of insulin injected and sticking to specific foods and portion sizes to stay well. The amount of sugar in the blood should also be measured regularly to make sure that levels are not too high or too low. We worked with large numbers of children age 6-18years to design three age appropriate diabetes self-management kits. The kits contained everything that the children said that they needed to better manage their diabetes. We then tested the new self-management kits in a large trial to see if children who used the kits were better able to manage their diabetes compared to those who did not use the kits. The trial showed that the kits made no difference and in some cases their diabetes management became worse. Over one third of children who were eligible did not want to take part in the trial. The kits caused some children to worry more and alienated both children and their parents. We asked children and their parents why they did not find the kits helpful. They told us that they did not like anything that reminded them that they had diabetes. Nor did they fully understand what good diabetes management involved or the risks associated with not managing their diabetes well. Children frequently reported that they did not like attending children's diabetes clinics or the authoritarian approach taken by diabetes professionals to their diabetes management. We concluded that there needs to be a fresh approach to the way that children's diabetes services are organised and managed. New ideas are needed about how best to design children's diabetes education.

For peer review only

Strengths and limitations of this study

- The self-management kits that were tested in this trial were designed with large numbers of children and young people and their parents in a 3 year study.
- We conducted a fully powered pragmatic trial of children's self-management kits in routine practice.
- A third of eligible children and young people declined to participate in the trial.

For peer review only - http://bmjopen.bmj.com/site/about/guidelines.xhtml

- Our large process evaluation provided a detailed explanation of the mechanisms that appear to lead to negative outcomes and lack of engagement by children and parents.
- The cost-effectiveness analysis was limited as there was no evidence of intervention effect.

For peer review only

Introduction

Managing diabetes at all ages costs the National Health Service (NHS) nearly £10 billion a year; 80% of this is for managing avoidable complications.[1] Learning to manage one's diabetes in childhood is important to prevent long-term and potentially life-threatening complications of poor glycaemic control. Diabetes care pathways[2-5] have been available for over a decade and the proportion of children in England and Wales who achieved the previous National Institute for Health and Care Excellence (NICE) target of an HbA_{1c} level of ≤58 mmol/mol has slowly increased from 14.5% in 2009 to 15.8% in 2013 and 26.6% in 2015.[6-8] In 2015 NICE further amended the target to ≤48mmol/mol.[9] Schools vary considerably in the support given to children to manage their insulin administration, diet, and participation in extracurricular activities and sports.[10] Achieving optimal glycaemic control is most difficult during transition from paediatric to adult services when young people become independent of parents and families.[11]

Optimal diabetes self-management requires titration of insulin doses against blood glucose levels, dietary intake and planned physical activities.[3,9] This skill is essential for children to participate fully in school life and social activities outside school.[10] Models of children's diabetes care emphasise a family-centred approach with intensive education and support following diagnosis, with increasing responsibility for care transferred to the child over time.[6-7] There has been no standardisation of diabetes self-management information given to children to use at home and school.

To prepare for the trial, research was undertaken in the current and a previous study with children, young people and parents to identify the types and formats of self-management information likely to inspire behaviour change in children and young people with diabetes.[12,13] Our systematic review of educational and support interventions to improve diabetes self-management in schools revealed no effective interventions, but many barriers to self-management when children were away from their parents.[10] Hence the goal of this trial was to evaluate whether standardised age-appropriate self-management kits motivate children and their families to avoid complications caused by uncontrolled blood sugar levels. Our primary aim was to assess whether the kits enabled children to manage their type 1 diabetes by titrating their insulin dose against regular blood glucose readings. Our secondary aim was to assess how children, their families and diabetes professionals perceived and used these kits.

For peer review only - http://bmjopen.bmj.com/site/about/guidelines.xhtml

The subsequent availability of published reports for five other contemporaneous trials of United Kingdom (UK) children's diabetes education interventions created a new opportunity to review all six trials and explore why none of these six interventions had any effect. In particular, since the original report was pubished in the NIHR journals library[12], we have now reanalysed HbA1c, a secondary outcome measure in our trial so that results can be discussed in relation to five contemporaneous UK trials and other international studies. We have also undertaken a more detailed analysis of the sub-domains of Diabetes PedsQL, the primary outcome measure of our trial, to better understand the benefits and harms of the intervention.

Methods

Study design and participants

We conducted a pragmatic randomised trial, including economic and process evaluations, in NHS paediatric diabetes clinics in England and Wales (ISRCTN 17551624).[12-14] The Wales Multi-Centre Research Ethics Committee gave approval (08/MRE09/57). The Medicines for Children Research Network (England) and the Children and Young People Research Network (Wales) recruited diabetes multi-disciplinary teams in 11 NHS District General Hospitals.

Intervention

Children in the comparator group received treatment as usually provided in each of the 11 diabetes clinics.[14] A record of 'usual care' was made in order to have a clear idea of the comparator with which EPIC was being compared. Children in the intervention group received a standardised but flexible self-management kit known as "Evidence into Practice – Information Counts" (EPIC). Following extensive literature review, consultation and fieldwork,[10,12-14] EPIC comprised:

- Three age-specific (6-10 years, 11-15 or 16-18) diabetes self-management kits comprising booklets, magazines, leaflets, CDs and website links;
- Three corresponding diaries for those using insulin injections*;
- One diary for children using insulin pumps*;
- Sheets for recording carbohydrate intake; and
- Stickers (6-15 years) and marker pens (11-15 years) for children to personalise their folder.

*children could also use blood glucose recordings downloaded from their blood glucose monitors.

For peer review only - http://bmjopen.bmj.com/site/about/guidelines.xhtml

A detailed account of intervention development and the theoretical basis of the EPIC intervention is reported elsewhere.[12] In brief, we designed EPIC so that children and young people had relevant information to self-manage their diabetes (with support from parents for younger children) consistent with relevant clinical guidelines,[2-5] incorporating age-appropriate preferences for information[13] and consistent with clinical practice in the UK NHS. Key features intended to appeal to children and engage them in EPIC included: presentation to and ownership by the child; age-appropriate messages stressing 'top ten tips' for self-management; the invitation 'take me with you wherever you go'; contextual questions about self-management and life-style; integration into routine encounters with the child's multi-disciplinary team, especially the Paediatrician and the Paediatric Diabetes Specialist Nurse (PDSN); scope for professionals and parents to tailor EPIC to each child and review at subsequent appointments; and encouraging the child to record blood glucose and insulin titration every day to share with professionals. We invited diabetes team members to attend training in their hospital which described EPIC, introduced the manual, and suggested how to engage each child, and how to integrate EPIC into routine care.

Random allocation

Between February 2010 and August 2011 we screened diabetes outpatient clinic lists in 11 hospitals for potentially eligible children between 6 and 18 years with type 1 diabetes. We excluded children with communication difficulties, needle phobia, or other impairments judged inconsistent with the trial. We sent invitation letters and age-specific information sheets to families of eligible children. Research nurses independent of both clinical and research teams sought written informed consent to the trial from parents and children over 16 years, or assent from children under 16 years. Consenting parents and their children provided baseline data.

Research nurses then used a secure web-based dynamic randomisation system[15] to allocate children at random between EPIC and treatment as usual, stratified by hospital, age, gender and whether two years had elapsed since diagnosis; the allocation ratio was two intervention participants for every control. These nurses told children's clinical teams of these allocations so they could initiate EPIC at the next consultation. We followed these children for 6 months.

Masking

For peer review only - http://bmjopen.bmj.com/site/about/guidelines.xhtml

Though it was neither desirable nor feasible to blind clinical staff or participants to treatment allocated, we sought to blind assessors. Analysis was undertaken by an independent trial support unit.

Outcome measures

The primary outcome was children's self-efficacy in coping with their diabetes, measured by child and (proxy) parent versions of the Diabetes PedsQL[16] six months after randomisation, with interim scores at three months. The Diabetes PedsQL comprises 33 items (32 for younger children) covering five domains – diabetes, treatment adherence, treatment barriers, communication and worry. The resulting scores lie between zero and 100 with higher scores indicating better coping.

Secondary outcomes comprised: HbA_{1c} measured at routine quarterly clinics; health-related quality of life measured by child and (proxy) parent versions of the General PedsQL;[17] and health-related quality of life measured by the EQ-5D-3L.[18] We used the youth version for children under 16 years, the adult version for those over 16, and parents' proxy scores for all children. Follow-up questionnaires, completed after three and six months in clinic or by post, also sought data on episodes of diabetic ketoacidosis and health service use, especially hospital admissions for acute complications, recorded on a diabetes-specific version of the Client Service Receipt Inventory.[19] We checked health service use against children's hospital notes.

Children and parents also completed baseline questionnaires covering socio-demographic characteristics and the duration and self-management of their diabetes. Children received £10 vouchers for each questionnaire they completed at 3 or 6 months. Non-responders received both telephone and postal reminders after two and four weeks.

We defined serious adverse events (SAEs) as adverse events that, in the judgement of the relevant site Principal Investigator, were lethal, life threatening, resulting in hospital admission, resulting in persistent or significant disability or incapacity, or otherwise medically significant. We defined serious adverse reactions (SARs) as SAEs that, in the judgement of the Clinical Principal Investigator (CPI) and research team, were definitely, probably or possibly related to the EPIC intervention or to treatment as usual.

Sample size

For peer review only - http://bmjopen.bmj.com/site/about/guidelines.xhtml

To yield 80% power of detecting an effect size of 0.4 in the primary outcome of self-efficacy when using a two-sided 5% significance level, we aimed to analyse 202 children, initially by recruiting 252 children – 168 allocated to EPIC and 84 controls, thus allowing for losing 20% of participants to follow up.[20,21] As fewer participants than expected initially responded to questionnaires, we reviewed these calculations in consultation with the Data Monitoring Committee and increased the target to 337 to allow for losing 40% to follow up. We also introduced monetary vouchers for completed questionnaires.

Statistical analyses

Analysis was by treatment allocated. We imputed missing quality of life data in accordance with published guidance for each measure. [22,23] We used the fully conditional specification technique and five multiple imputations across time points to impute these data.[22] We compared differences between treatment groups using mixed models to undertake repeated-measures analysis of variance, adjusting for stratification variables and baseline values. We estimated parameters for three fixed factors – the time-points of three and six months and treatment group. We modelled hospital as a random factor. We included the interaction between treatment group and time-point to test whether differences between treatment groups varied between time-points. These analyses modelled diabetes self-efficacy (Diabetes PedsQL), quality of life (General PedsQL) and health utility (EQ-5D), both to study change in individuals, and in cohort analysis to compare change in group means.

Economic analysis

We costed the age-specific EPIC kits by recording quantities and costs of materials used to produce them, and estimating the mean additional time taken by PDSNs. We collected retrospective data on children's use of primary and secondary healthcare services over the previous three months. We applied national unit costs in 2010-11 pounds sterling (£) to these services.[24,25] As we followed participants for only six months, we did not discount costs or effects.[26] We undertook cost-consequence analysis from an NHS perspective and tested the sensitivity of findings to the substitution of consultants for nurses in presenting the EPIC kits.

Process evaluation

After the trial we recruited a second sample for semi-structured recorded interviews in depth. The process evaluation was conducted up to December 2013. We used purposive sampling to

For peer review only - http://bmjopen.bmj.com/site/about/guidelines.xhtml

generate maximum variation in the ages, genders, times since diagnosis, and types of insulin transfer (injection or pump) of 41 children allocated to EPIC, and 19 comparator children. We also interviewed 66 parents and family members of these children. These interviews explored: views and experiences of both EPIC kits and treatment as usual; how participants managed self-care at home and in school and other social contexts; and children's interactions with diabetes teams. Before the trial we interviewed professionals in each of the 11 hospitals about their previous practice; after the trial we surveyed them by post about how they had implemented EPIC. We recorded interviews, transcribed them verbatim, and analysed them using the thematic framework approach.[27] We mapped the resulting themes onto the underlying theory and developed higher level themes and understanding in depth.

As part of the process evaluation we also undertook a discourse analysis of a purposive sample of children's diabetes resources used in the UK NHS.[28] Discourse analysis is a way of identifying and analysing the assumptions made by information sources about their relationships with their readership, assumptions that had shaped the messages of the selected resources, some of which had appeared in the intervention pack and may also have been available to comparator children.

Patient and public involvement

Children, parents and public representatives were actively involved in an extensive three year prior study, as well as the current study to develop the educational intervention.[12] A core set of diabetes education materials were co-designed by children with type 1 diabetes of various ages. Children and parents were represented in the trial advisory group. All participants received a child-centred copy of the findings.

Results

Figures 1 and 2 show the flow of participants through the study. We screened 1105 children identified as potentially eligible from the clinic lists of 11 participating hospitals: 146 (13.2%) were not eligible by the trial criteria, 335 (30.3%) declined to participate after receiving letters of invitation and trial information sheets, 287 (26.0%) were missed in clinic or did not join the trial for other reasons. Hence we randomised 337 children.

Insert Figures 1 and 2

For peer review only - http://bmjopen.bmj.com/site/about/guidelines.xhtml

Response rates

Two protocol violations affected 29 children. More importantly 21 intervention and 7 comparator children did not complete baseline questionnaires before receiving treatment within the trial, most because one centre allowed them to take questionnaires home for return by post. The other violation randomised the same child twice following a change of web servers at another site. Fortunately sensitivity analysis including these 29 children showed essentially the same results.[15] Figure 2 shows that, of the 308 fully compliant children, 256 (84%) returned questionnaires at 3 months, 266 (86%) did so at 6 months and imputation enabled us to analyse 293 (95%). Thus the incentive of shopping vouchers achieved much higher response rates than our targets. Hence the trial was better powered than planned.

Baseline characteristics

Table 1 shows characteristics of the 293 analysable participants at baseline.

Table 1: Characteristics of participants at baseline by allocated group

		EPIC kit	Treatment as usual
	**N (row %)**	190 (65%)	103 (35%)
DEMOGRAPHIC CHARACTERISTICS [n (%) unless specified]			
**Gender**	Male	85 (45)	49 (48)
	Female	105 (55)	54 (52)
**Age in years**	Range	6.3-18.9	6.4-18.4
	Mean (SD)	12.4 (3.0)	12.7 (3.2)
**Ethnicity**	White British	179 (94)	101 (98)
	Other	11 (6)	2 (2)
**Education & employment**	Secondary school	13 (42)	7 (33)
	Further Education College	13 (42)	12 (57)
	Other	5 (16)	1 (5)
	Age <16 so not asked	159	86
**Living situation**	Owner occupied house/flat	156 (82)	86 (83)
	Privately rented house/flat	20 (11)	6 (6)
	Housing assoc./local authority	14 (7)	11 (11)
**Years since diagnosis**	Range	0.8 – 16.7	1.2 – 15.7
	Mean (SD)	7.4 (3.8)	8.0 (3.9)
**Type of insulin administration**	Injections	167 (88)	87 (84)
	Pump	23 (12)	16 (16)
**Insulin regimen**	Once a day	2 (1)	-
	2 times a day	41 (25)	19 (22)

For peer review only - http://bmjopen.bmj.com/site/about/guidelines.xhtml

	3 times a day	14 (8)	10 (11)
	4 times a day	95 (57)	45 (52)
	Other (at least 5 times a day)	15 (9)	13 (15)
**Blood glucose tests**	None	1 (1)	1 (1)
	Once a day	4 (2)	1 (1)
	2 times a day	7 (4)	5 (5)
	3 times a day	31 (16)	16 (16)
	4 times a day	89 (47)	50 (49)
	Other (at least 5 times a day)	57 (30)	29 (28)
	Missing	1	1
**HbA_{1C} (%)** **(*mmol/mol*)** [1]	Range	5.9-14.0 *41.0-129.5*	6.0-13.7 *42.1-126.2*
	Mean	8.77/*72.3*	8.59/*70.4*

For peer review only - http://bmjopen.bmj.com/site/about/guidelines.xhtml

QOL BASELINE MEASURES AT TRIAL ENTRY			
Child self-report	PedsQL: Diabetes module (total scale score 100)	73.63 (14.68)	73.29 (12.17)
	PedsQL: Generic module (total scale score 100)	83.70 (12.36)	81.78 (12.63)
	EQ-5D utility score (total scale score 1)	0.9012 (0.1501)	0.8976 (0.1537)
	EQ-5D VAS (total scale score 100)	83.22 (16.98)	77.86 (18.89)
Parent proxy	PedsQL: Diabetes module (total scale score 100)	65.82 (15.35)	65.65 (14.02)
	PedsQL: Generic module (total scale score 100)	77.86 (14.66)	77.78 (14.43)
	EQ-5D utility score (total scale score 1)	0.8499 (0.1733)	0.8231 (0.1800)
	EQ-5D VAS (total scale score100)	83.02 (16.40)	79.96 (19.11)

[1] Pooled estimates.

Primary outcome

Tables 2 and 3 show that, of the five dimensions of the Diabetes PedsQL, only Worry showed adjusted scores significantly favouring self-management kits at three months: the mean child-reported difference was +5.87 with standard error [SE] of 2.19, generating statistical significance level (p) of 0.8% and a 95% confidence interval [CI] from +1.57 to +10.18. At six months, however, only Treatment Adherence achieved significance – in favour of treatment as usual: the mean child-reported difference was –4.68 with SE of 1.74, generating another p of 0.8% and 95% CI from –8.10 to –1.25. Even worse, intervention children reported significant adverse changes between three and six months on four of the five Diabetes PedsQL dimensions and thus on the total score: the mean child-reported difference was –3.20 with statistical significance level of 2.0% and 95% CI from –5.73 to –0.67.

Secondary outcomes

Moreover there was no evidence of change in HbA_{1c}. Participants started the trial with a mean baseline HbA_{1c} of 72 mmol/mol. Whether adjusted by baseline scores and stratification variables or not, this mean remained virtually unchanged during the follow up period: after three months self-managing children had reduced their adjusted HbA_{1c} by only –0.63 mmol/mol relative to comparator children (p=0.64; 95% CI from –3.31 to +2.04; after six months the reduction was only –0.19 mmol/mol (p=0.90; 95% CI from –3.07 to +2.70). At baseline only 16% of children across both treatment groups had achieved HbA_{1c} levels ≤ 58 mmol/mol, and at six months only 18%, thereby matching the national average in 2012/3.[5]

For peer review only - http://bmjopen.bmj.com/site/about/guidelines.xhtml

Tables 2 and 3 also show that the only statistically significant difference between groups in General PedsQL or EQ-5D-3L was that self-managing children reported that their School Functioning after six months was worse by –5.79 on average ($p < 0.001$; 95% CI from –9.21 to –2.36). Those intervention children also reported significant adverse changes between three and six months on two of the four Diabetes PedsQL dimensions and thus on the total score: the mean child-reported difference was –5.78 (p=0.002; 95% CI from –9.39 to –2.17).

However the parent-reported PedsQL scores, showed a different pattern from the child-reported scores: no delayed negative effects were apparent; instead comparator children were significantly better after three months in Diabetes Symptoms by –2.93 (p=0.036; 95% CI from –5.68 to –0.19); and in Treatment Adherence by –3.86 (p=0.026; 95% CI from –7.25 to –0.47).

Adverse events

Participating clinicians reported 31 serious adverse events to the trial team – 22 in the intervention group and 9 in the comparator group, yielding a relative risk of 1.33 (p=0.45; 95% CI from 0.63 to 2.77). The CPI, and the Chairs of the Data Monitoring & Ethics Committee and the Trial Steering Committee, reviewed and confirmed these SAEs. The CPI and research team judged that 4 SAEs from the Intervention Group and 2 from the Control Group were SARs possibly related to the EPIC intervention or to treatment as usual, yielding a relative risk of 1.08 (p=0.92; 95% CI from 0.20 to 5.82).

For peer review only - http://bmjopen.bmj.com/site/about/guidelines.xhtml

Table 2: Mixed models adjusted by stratification variables and baseline values BY treatment allocated

Outcome variable	Difference (Epic Pack– Treatment as usual)					Covariates/Cofactors sig at 5%		
	F(1,290)	p	Mean	s.e.	95% CI		F(1,290)	p
**Child self-report**								
**PedsQL: General**								
Total score	0.76	0.384	-0.96	1.10	-3.13 to 1.21	Baseline	275	<0.001
						Time-point	4.36	0.038
						Treatment group by time-point	5.13	0.024
Physical functioning	0.00	0.974	0.04	1.32	-2.56 to 2.65	Baseline	103	<0.001
						Time-point	5.38	0.021
						Treatment group by time-point	8.14	0.005
Emotional functioning	0.08	0.780	-0.51	1.83	-4.12 to 3.09	Baseline	184	<0.001
Social functioning	0.00	0.971	-0.05	1.46	-2.92 to 2.81	Baseline	181	<0.001
School functioning	4.26	0.040	-2.90	1.40	-5.66 to -0.14	Baseline	271	<0.001
						Treatment group by time-point	9.59	0.002
**PedsQL: Diabetes**								
Total score	0.07	0.798	-0.32	1.26	-2.80 to 2.16	Baseline	183	<0.001
						Treatment group by time-point	5.44	0.020
Diabetes symptoms	0.00	0.955	-0.09	1.55	-3.14 to 2.96	Baseline	170	<0.001
Treatment barriers	0.02	0.876	-0.27	1.71	-3.64 to 3.10	Baseline	174	<0.001
						Treatment group by time-point	4.19	0.042
Treatment adherence	2.60	0.108	-2.38	1.47	-5.28 to 0.52	Baseline	78	<0.001
						Treatment group-by-time-point	6.87	0.009
Worry	2.76	0.098	3.23	1.94	-0.60 to 7.05	Baseline	176	<0.001
						Treatment group by time-point	4.88	0.028
Communication	0.13	0.720	0.69	1.93	-3.10 to 4.48	Baseline	107	<0.001
						Treatment group-by-time-point	7.17	0.008
**EQ-5D**	0.00	0.960	.001	.018	-.034 to .036	Baseline	115	<0.001
						Gender	6.12	0.014

For peer review only - http://bmjopen.bmj.com/site/about/guidelines.xhtml

For peer review only

						Length of time since diagnosis	6.82	0.009
**EQ-5D: VAS**	0.42	0.520	1.10	1.70	-2.26 to 4.45	Baseline	125	<0.001
						Age	4.05	0.045
**Parent proxy**								
**PedsQL: General**								
Total score	0.66	0.417	0.94	1.15	-1.33 to 3.20	Baseline	264	<0.001
Physical functioning	2.61	0.107	2.22	1.38	-0.48 to 4.93	Baseline	94	<0.001
Emotional functioning	0.06	0.799	-0.48	1.86	-4.15 to 3.20	Baseline	143	<0.001
Social functioning	0.25	0.620	0.72	1.45	-2.13 to 3.57	Baseline	292	<0.001
						Age	5.28	0.022
School functioning	0.463	0.497	0.97	1.42	-1.84 to 3.77	Baseline	316	<0.001
						Time point	6.81	0.010
**PedsQL: Diabetes**								
Total score	2.36	0.125	-1.68	1.09	-3.82 to 0.47	Baseline	412	<0.001
Diabetes symptoms	1.44	0.232	-1.55	1.29	-4.09 to 1.00	Baseline	390	<0.001
Treatment barriers	0.57	0.452	-1.20	1.60	-4.36 to 1.95	Baseline	270	<0.001
						Age	5.67	0.018
Treatment adherence	6.15	0.014	-3.48	1.40	-6.23 to -0.72	Baseline	218	<0.001
Worry	0.15	0.697	-0.77	1.98	-4.66 to 3.12	Baseline	200	<0.001
						Time-point	9.49	0.002
Communication	0.11	0.742	0.67	2.04	-3.34 to 4.69	Baseline	171	<0.001
**EQ-5D**	1.82	0.178	-0.025	0.019	-0.062 to 0.012	Baseline	71	<0.001
**EQ-5D: VAS**	0.76	0.385	-1.34	1.54	-4.38 to 1.69	Baseline	171	<0.001
						Gender	5.22	0.023
**HbA1c**								
mmol/mol	0.11	0.740	-0.40	1.22	-2.80 to 1.99	Baseline	330	<0.001
	**F(1,290)**	**p**	**Mean**	**s.e.**	**95% CI**		**F(1,290)**	**p**

For peer review only - http://bmjopen.bmj.com/site/about/guidelines.xhtml

For peer review only

Table 3: Mixed models: mean effect at 3 & 6 months estimated from main model

Outcome variable	Time point	Difference (Epic Pack– Treatment as usual)					
		Mean	s.e.	95% CI	p	Change (6mth-3mth)	p
**Child self-report**							
**PedsQL: General**							
Total score	3mth	0.52	1.26	(-1.96 to 2.99)	0.682	-2.96	0.024
	6mth	-2.44	1.30	(-5.01 to 0.13)	0.063		
Physical functioning	3mth	2.16	1.59	(-0.96 to 5.29)	0.174	-4.24	0.005
	6mth	-2.08	1.44	(-4.92 to 0.76)	0.151		
Emotional functioning	3mth	-0.60	2.06	(-4.65 to 3.45)	0.769	0.18	0.932
	6mth	-0.42	2.19	(-4.73 to 3.89)	0.848		
Social functioning	3mth	0.55	1.62	(-2.64 to 3.75)	0.734	-1.21	0.455
	6mth	-0.66	1.72	(-4.03 to 2.72)	0.701		
School functioning	3mth	-0.01	1.63	(-3.21 to 3.20)	0.997	-5.78	0.002
	6mth	-5.79	1.74	(-9.21 to -2.36)	0.001		
**PedsQL: Diabetes**							
Total score	3mth	1.23	1.39	(-1.50 to 3.96)	0.375	-3.11	0.020
	6mth	-1.88	1.46	(-4.76 to 1.00)	0.200		
Diabetes symptoms	3mth	0.14	1.72	(-3.26 to 3.53)	0.938	-0.45	0.803
	6mth	-0.31	1.81	(-3.88 to 3.26)	0.865		
Treatment barriers	3mth	1.77	1.91	(-1.98 to 5.52)	0.355	-4.07	0.042
	6mth	-2.30	2.05	(-6.34 to 1.74)	0.263		
Treatment adherence	3mth	-0.08	1.69	(-3.40 to 3.25)	0.964	-4.76	0.009
	6mth	-4.68	1.74	(-8.10 to -1.25)	0.008		
Worry	3mth	5.87	2.19	(1.57 to 10.18)	0.008	-5.29	0.028
	6mth	0.58	2.37	(-4.09 to 5.25)	0.808		
Communication	3mth	3.66	2.10	(-0.47 to 7.80)	0.082	-5.94	0.008
	6mth	-2.28	2.34	(-6.89 to 2.32)	0.330		
**EQ-5D**	3mth	0.013	0.023	(-0.032 to 0.059)	0.568	-0.025	0.304
	6mth	-0.011	0.019	(-0.050 to 0.027)	0.555		
**EQ-5D: VAS**	3mth	0.58	1.83	(-3.02 to 4.18)	0.751	1.04	0.569
	6mth	1.62	2.03	(-2.37 to 5.60)	0.426		
**Parent proxy**							
**PedsQL: General**							
Total score	3mth	0.04	0.976	(-2.43 to 2.51)	0.976	1.79	0.114
	6mth	1.83	0.191	(-0.92 to 4.58)	0.191		
Physical functioning	3mth	1.53	1.53	(-1.49 to 4.55)	0.319	1.39	0.399
	6mth	2.92	1.66	(-0.36 to 6.19)	0.081		
Emotional functioning	3mth	-2.44	2.10	(-6.58 to 1.71)	0.248	3.93	0.068
	6mth	1.49	2.20	(-2.84 to 5.81)	0.499		
Social functioning	3mth	1.16	1.60	(-1.99 to 4.30)	0.470	-0.88	0.586
	6mth	0.28	1.71	(-3.09 to 3.65)	0.870		
School functioning	3mth	-0.53	1.72	(-3.91 to 2.85)	0.757	3.00	0.114

For peer review only - http://bmjopen.bmj.com/site/about/guidelines.xhtml

	6mth	2.47	1.70	(-0.88 to 5.82)	0.148		
**PedsQL: Diabetes**							
Total score	3mth	-2.29	1.20	(-4.64 to 0.07)	0.057	3.49	0.781
	6mth	1.20	1.38	(-3.78 to 1.65)	0.440		
Diabetes symptoms	3mth	-2.93	1.40	(-5.68 to -0.19)	0.036	2.77	0.074
	6mth	-0.16	1.61	(-3.33 to 3.01)	0.920		
Treatment barriers	3mth	-2.71	1.85	(-6.36 to 0.94)	0.145	3.01	0.145
	6mth	0.30	1.98	(-3.59 to 4.19)	0.880		
Treatment adherence	3mth	-3.86	1.72	(-7.25 to -0.47)	0.026	0.77	0.702
	6mth	-3.09	1.73	(-6.50 to 0.32)	0.076		
Worry	3mth	1.15	2.44	(-3.64 to 5.94)	0.637	3.84	0.161
	6mth	-2.69	2.38	(-7.37 to 1.98)	0.258		
Communication	3mth	1.04	2.43	(-3.74 to 5.83)	0.668	1.98	0.768
	6mth	3.02	2.36	(-4.34 to 4.94)	0.898		
**EQ-5D**	3mth	-0.024	0.023	(-0.069 to 0.020)	0.280	-0.001	0.963
	6mth	-0.026	0.022	(-0.070 to 0.019)	0.254		
**EQ-5D: VAS**	3mth	-2.00	1.78	(-5.50 to 1.51)	0.263	1.31	0.484
	6mth	-0.69	1.82	(-4.28 to 2.90)	0.706		
**HbA1c**							
mmol/mol	3mth	-0.63	1.36	(-3.31 to 2.04)	0.641	0.45	0.765
	6mth	-0.19	1.46	(-3.07 to 2.70)	0.899		

Sensitivity analyses

Our sensitivity analysis confirmed that participants with incomplete outcomes (withdrawals or those lost to follow up) did not change the findings of this trial.

Post-hoc analysis

Given the shortage of treatment effects and the varying size of the three age subgroups, we undertook post-hoc analysis to see if there was any evidence of an effect within individual age groups bands. We found no evidence of differences in outcomes by age group.

Cost consequences

We based economic analysis on 233 (80%) of the 293 children in the effectiveness analysis. We excluded 60 (20%) children with incomplete data on costs and service use because we could not be sure that these were missing at random. The mean total intervention unit cost of producing and administering intervention kits was £185 (Table 4A). The mean total cost (NHS costs including intervention kit and administration costs) was £136 (bootstrapped 95% CI: -£52, £296) higher for the intervention group than for the comparator group, but this

For peer review only

For peer review only - http://bmjopen.bmj.com/site/about/guidelines.xhtml

difference was not statistically significant (Table 4B). We undertook bootstrapping with 1000 replicates to estimate a 95% confidence interval around this mean difference in costs and consequences between groups. For consequences, there were no significant mean differences for any outcome. Sensitivity analysis postulating that consultants instead of PDSNs see children in clinic but for the same 13.2 minutes, increased the mean difference in total costs of service use between groups from £136 to £182 (bootstrapped 95% confidence interval: -£9 to £339), but this difference was still not statistically significant.

For peer review only

For peer review only - http://bmjopen.bmj.com/site/about/guidelines.xhtml

Table 4A. Costs of producing and distributing self-management intervention kits and distributing them to participants by PDSNs in clinics in 2011 UK pounds (£)

Age group	6-10 years		11-15 years		16-18 years		Across all ages
Administration route	Injection	Pump	Injection	Pump	Injection	Pump	
Cost of self-management kit [a] (£)	11.57	16.67	12.09	17.19	22.07	28.29	
Cost of extra PDSN appointments [b] (£)	166.66	166.66	166.66	166.66	166.66	166.66	
**Total cost per participant (£)**	**178.23**	**183.33**	**178.75**	**183.85**	**188.73**	**194.95**	**184.64**
**Number in Intervention Group**	49	9	66	8	21	5	158
**Total cost of intervention (£)**	**8733**	**1650**	**11798**	**1471**	**3963**	**975**	**28590**
**Average cost per participant (£)**							**180.95**

Notes:

a. Including 'treatment cost' of producing and printing age-specific diabetes diaries, but not 'research cost' of developing these diaries.
b. Including 'treatment cost' of estimated time of Paediatric Diabetes Specialist Nurses (PDSNs) in teaching children about the kit, but not 'research cost' of research nurses distributing the kit.

For peer review only

Table 4B. Health service use costs and consequences by allocated group over 6 months

	Intervention kits (n=158) Mean (SD)	Treatment as usual (n=75) Mean (SD)	Intervention minus treatment as usual (bootstrapped 95% CIs)
Costs			
**Primary care (£)** [a]	67 (111)	61 (90)	6 (-22, 32)
**Secondary care (£)** [a]	454 (524)	504 (648)	-50 (-226, 103)
**EPIC intervention cost (£)** [a, b]	181 (4)	0 (0)	181
**Total cost / participant (£)**	702 (558)	566 (664)	136 (-52, 296)
Consequences			
Participant self-report:			
**QALYs**	0.446 (0.0741)	0.447 (0.0784)	-0.001 (-0.0209, 0.0189)
Parent-proxy:			
**QALYs**	0.415 (0.0785)	0.418 (0.0831)	-0.003 (-0.0238, 0.0188)

Notes:
[a] Mean (SD) total cost per participant (£)
[b] Cost of intervention includes: the 'treatment costs' of producing and distributing the kit to participants by Paediatric Diabetes Specialist Nurses in clinics; those of producing and distributing the intervention diabetes diary; but not the 'research costs' of developing kit or diary.

For peer review only - http://bmjopen.bmj.com/site/about/guidelines.xhtml

For peer review only

Process Evaluation

We present key findings from the process evaluation [12] to illustrate the wider context within which children were initially excited but thereafter did not engage as intended with diabetes self-management generally or use the standardised kits specifically. We offer an explanation as to how the context was created for this mechanism to occur.

Normalisation in children's self-management and relationships with professionals

After initial excitement, most children said that they did not use the EPIC kits as intended; a few rejected them completely and put them out of sight, for example in the loft. Analysis of the words, messages and images in children's self-management information found that they generally presented rules to manage diabetes supported by images of being 'normal' like other children if they followed those rules and did what professionals said. The process evaluation showed that these authoritarian normalisation messages did not always resonate with children, especially teenagers, as they did not feel normal because their life was frequently defined by diabetes, which they disliked. The presence of the EPIC kits and the messages they contained caused increasing levels of worry and anxiety. So they tried to hide diabetes by not making 'self-management' visible to themselves or others. Hence few children took a diabetes diary to school or wanted to test their blood sugar levels.

Children's inability to associate blood glucose tests with better management

Baseline questionnaires from 308 children and their parents at entry to the trial show that they knew how many times a day they should record their blood glucose. In reality most children, especially teenagers and irrespective of allocation, did not use or even see the need to record or observe trends in blood glucose levels to titrate their insulin dose. Of those interviewed in the intervention treatment group, around half of 6-10 year olds, less than half of 11-15 year olds, but only around 20% of 16-18 year olds said that they or their parents recorded blood glucose levels; fewer still appeared to use levels to titrate insulin doses. Children more commonly neglected the age-appropriate self-management information provided. Many children thought that they were recording this information for the benefit of diabetes professionals and made no link between blood glucose testing and gaining better diabetes control.

For peer review only - http://bmjopen.bmj.com/site/about/guidelines.xhtml

Children's ignorance of risky behaviour and long-term complications of diabetes

By not titrating insulin doses to blood glucose levels, many children took risks with their diabetes-related health; many teenagers appeared unconcerned about the potential consequences. Some parents said they wanted to protect their children from receiving information on risks and complications, whereas others wanted to expose their children to the reality of life threatening complications like renal failure. Discourse analysis showed that: children's diabetes information resources for ages 6-10 years rarely mentioned risks or complications of poorly controlled diabetes; while those for ages 11-15 years were usually vague about serious risks and long-term complications of poorly controlled diabetes. In contrast information distributed on entry to adult diabetes services was explicit about risks of long-term complications and the resulting need for self-management to minimise these.

Promotion of intervention by diabetes professionals and parents

Most diabetes teams did not actively engage with the intervention kits or encourage their use in routine consultations. Around a third of children approached declined to participate. Often there were modifications to intended intervention delivery; for example the research nurse, not a member of the child's diabetes team, gave the kit and diary to the child. There was little individualisation or tailoring of intervention kits. Around half the PDSNs regulated the information given to children and knowingly withheld or removed information on lifestyle issues and risks of complications before distribution to children. From the kit for ages 11-15 distributed to 103 participants, professionals reported removing several topics as inappropriate: sex and beyond (12 times), drinking alcohol (11 times), body piercing (8 times), and carbohydrate awareness (4 times). After children had received the kits, many parents had removed anything they thought unsuitable, notably the 'lifestyle' resources, if not already removed by the PDSN.

For peer review only - http://bmjopen.bmj.com/site/about/guidelines.xhtml

Discussion

Principal findings

This pragmatic trial found no evidence of benefit from age-appropriate diabetes self-management kits for children. Of the five dimensions of the Diabetes PedsQL, Worry showed adjusted scores significantly favouring self-management kits at three months but Treatment Adherence significantly favoured controls at six months. Furthermore children using self-management kits reported significantly worse changes between three and six months on four of the five Diabetes PedsQL dimensions and thus on the total score. There was no evidence of change in HbA1c; only 18% of participants in each group achieved recommended levels at 6 months. This was apparently because the kits alienated children and parents, and their use of kits and recording of blood glucose was poor. Moreover five [29-33] other contemporaneous UK trials of educational interventions for childhood diabetes reported no benefit and little rapport between children, their parents, clinic staff, and the interventions. The other five interventions comprised structured diabetes education, family-based diabetes education and support [30-33] and training in communication skills[29]. None of these six UK trials (including EPIC) showed any difference in HbA_{1c} between groups; or evidence of any other benefit. EPIC is the first trial to make clear a deleterious intervention effect over time. As fewer than 20% of all 2018 children across the six trials achieved glycaemic control (pre 2015 HbA1c target of ≤58 mmol/mol), this meant that over 80% of children were at risk of serious complications. All six trials reported concerns about intervention fidelity. Attendance at additional diabetes teaching sessions was highly variable, and those with the highest HbA_{1c} were least likely to attend. A recent systematic review of ten trials of education and psychoeducation interventions (Including the five trials above but excluding EPIC as HbA1c had not been fully analysed at the time) showed a non-significant reduction in HbA1c attributable to the intervention (pooled SMD = -0.06, 95% CI: -0.21 to 0.09).[34]

Our parallel analysis of the words and images in diabetes resources provides new insights into the potential source of the surprisingly adverse effects of the kits. These stem in part from children's rejection of 'unwelcome' information which labelled them as different in having diabetes and authoritarian instructions like 'take me with you wherever you go'.

For peer review only

More generally the discourse of 'normalisation' through optimal management and insulin as a social enabler appears counterproductive in promoting desired behaviour change. Poor relationships between children, parents and diabetes professionals is another reason for lack of 'compliance' with diabetes professionals' expectations. In short diabetes communicators do not yet know how to convey effective messages to children. One trial (DEPICTED)[29], which attempted to train health-care professionals do this, also failed to show any benefit.

Strengths and weaknesses of this study

We based the EPIC kit on extensive research with children, parents and professionals; and met international standards for patient information.[12-13] We powered the EPIC trial to detect plausible effect sizes for the entire age-range, and surpassed recruitment targets.[20,21] Particular strengths included the preparatory discourse analysis of children's diabetes self-management information and the process evaluation in which children assessed the ways in which they received key diabetes self-management information. In retrospect the major weakness was the unpopularity of the EPIC kit.

Interpretation

Lack of progress in meeting the NICE target for HbA_{1c} in children stimulated the commissioning of this and five other contemporaneous trials by UK funders to test various approaches to promote optimal self-management. Other promising developments since the delivery of this trial, include electronic management systems, diabetes phone and iPad applications, and personalised web-based diabetes training programmes.[27-30] However these are unlikely to benefit children if, despite receiving good information, they still do not make the link between blood glucose testing and achieving glycaemic control through insulin, diet and lifestyle management.

In contrast, international studies show that children can achieve acceptable glycaemic control through investment in structured education and patient management following diagnosis, with outcomes ranging from 29% to over 50% of children achieving an HbA1c level less than ≤58mmol/mol [35-36] this is much higher than the 18% achieved by EPIC & the UK in general. The Hvidøre Study Group [35] identified management from the diagnosis of the

For peer review only - http://bmjopen.bmj.com/site/about/guidelines.xhtml

disease, positive and shared attitudes within diabetes teams and greater patient empowerment as factors that enhanced glycaemic control. Though we understand these factors, we do not know why UK children cannot achieve similar standards.

Implications for practice and research

Our process evaluation and discourse analysis identified two problems which have not yet been adequately addressed. First, we need to understand better how children respond to 'authority' in relationships with diabetes professionals. Expected regular attendance at outpatient clinics, the normative nature of most self-management information, and the withholding of requested lifestyle information all reinforce the power imbalance. Despite the intended focus on 'normalisation', children feel different, dislike being different, and tend to reject any intervention that reminds them of that difference. The second problem is that children, parents and professionals cannot reconcile clinical risk definitions and personal ones. Children assess risks differently from diabetes professionals and take rational decisions about what is acceptable for them. To overcome this, professionals may need to address risk collaboratively rather than hierarchically and this would require a complete rethink as to how diabetes services are designed and delivered. Motivational interviewing has, for example, been shown to be an effective method of facilitating behavioural changes in teenagers with type 1 diabetes with subsequent improvement in their glycaemic control.[37]

There are also implications for trial methods and conduct. First, we need to encourage research and clinical teams to implement complex interventions like this rigorously but also flexibly. Secondly, as the care of children's diabetes varies widely across the UK, there is merit in engaging the diabetes teams likely to contribute to a planned trial in design and planning.

For peer review only

References

1. NHS Digital. National diabetes audit, 2015-16 report 2a: Complications and mortality (complications of diabetes) England and Wales 2017:https://files.digital.nhs.uk/pdf/4/t/national_diabetes_audit__2015-16__report_2a.pdf.

2. International Society for Pediatric and Adolescent Diabetes. ISPAD clinical practice consensus guidelines 2009. www.ispad.org/resource-type/ispad-clinical-practice-consensus-guidelines- 2009. Accessed 10.3, 2013.

3. National Institute for Health and Care Excellence. Type 1 diabetes: Diagnosis and management of type 1 diabetes in children and young people. *London: National Institute for Clinical Excellence*. 2004.

4. Royal College of Paediatrics and Child Health (RCPCH). Growing up with diabetes: Children and Young people with diabetes in ENGLAND. . *RCPCH, London.* 2009.

5. Scottish Intercollegiate Guidelines Network. Management of diabetes: A national clinical guideline. 2010;116.

6. NHS Information Centre. National diabetes paediatric audit report 2009–10. *Leeds: NHS Information Centre for Health and Social Care; URL: www.diabetes.org.uk/Documents/ News/Paediatric_Diabetes_Audit130711.pdf*. 2011.

7. NHS Information Centre. National diabetes paediatric audit report 2011–12. *Leeds: NHS Information Centre for Health and Social Care http://www rcpch ac uk/child-health/standards-care/clinical-audit-and-quality-improvement/national-paediatric-diabetes-au-1*. 2012.

8. NHS Information Centre. National paediatric diabetes audit 2012-13. . 2013;http://www.rcpch.ac.uk/system/files/protected/page/NPDA%202012-13%20Core%20Report%202nd%20FINAL%20v%203.3.pdf.

9. NICE National Institute for Health and care Excellence. Diabetes (type 1 and type 2) in children and young people: Diagnosis and management. . 2015;https://www.nice.org.uk/guidance/ng18/resources/diabetes-type-1-and-type-2-in-children-and-young-people-diagnosis-and-management-pdf-1837278149317.

10. Edwards D, Noyes J, Lowes L, Haf Spencer L, Gregory JW. An ongoing struggle: A mixed-method systematic review of interventions, barriers and facilitators to achieving optimal self-care by children and young people with type 1 diabetes in educational settings. *BMC Pediatr*. 2014;14:228-doi: 10.1186/1471-2431-14-228.

11. Allen D, Channon S, Cohen D, Hooten N, Lowes L, Owens D, et al. The transition from paediatric to adult diabetes services: What works, for whom and in what circumstances? final report. *NIHR Service Delivery and Organisation Programme*. 2010.

12. Noyes JP, Lowes L, Whitaker R, Allen D, Carter C, Edwards RT, et al. Developing and evaluating a child-centred intervention for diabetes medicine management using mixed methods and a multicentre randomised controlled trial. *Health Serv Deliv Res*. 2014;2(8):1-482.

13. Williams A, Noyes J, Chandler-Oatts J, Allen D, Brocklehurst P, Carter C, et al. Children's health information matters: Researching the practice of and requirements for age appropriate health information for children and young people. final report. *NIHR Service Delivery and Organisation Programme.* 2011.

For peer review only - http://bmjopen.bmj.com/site/about/guidelines.xhtml

14. Noyes J, Williams A, Allan D, Brocklehurst P, Carter C, Gregory J, et al. Evidence into practice: Evaluating a child-centred intervention for diabetes medicine management: The EPIC project. *BMC Pediatrics*. 2010;10(70):3-12.

15. Russell D, Hoare Z, Whittaker R, Whittaker C, Russell I. Generalized method for adaptive randomization in clinical trials. *Stat Med*. 2010;30:922-924.

16. Varni J, Burwinkle T, Jacobs J, Gottschalk M, Kaufman F, Jones K. The PedsQLTM in type 1 and type 2 diabetes. reliability and validity of the pediatric quality of life InventoryTM generic core scales and type 1 diabetes module. *Diabetes Care*. 2003;26:631-637.

17. Varni JW, Burwinkle TM, Seid M. The PedsQL™ as a pediatric patient-reported outcome: Reliability and validity of the PedsQL™ measurement model in 25,000 children. . *Expert Rev Pharmacoecon Outcomes Res*. 2005;5:705-719.

18. Euroqual. Eq-5d. http://www.euroqol.org/2018.

19. Beecham J, & Knapp M. Costing psychiatric interventions. In: Thornicroft G, Brewin C, Wing J., ed. *Measuring mental health needs.* Oxford: Oxford University Press; 1992:200-224.

20. Murphy H, Rayman G, Skinner T. Psycho-educational interventions for children and young people with type 1 diabetes. *Diabet Med*. 2006;23:935-943.

21. Hampson S, Skinner T, Hart J, Storey L, Gage H, Foxcroft D, et al. Effects of educational and psychosocial interventions for adolescents with diabetes mellitus: A systematic review . *Health Technol Assess*. 2001;5(10)(10).

22. Van Buuren S, Brand JPL, Groothuis-Oudshoorn K, Rubin D. Fully conditional specification in multivariate imputation. *Journal of Statistical Computation and Simulation*. 2006;76(12):1049-1064.

23. Khutoryansky NM HW. Imputation techniques using SAS software for incomplete data in diabetes clinical trials. 2001:335-359.

24. Curtis L. Unit costs of health and social care 2011. https://www.pssru.ac.uk/pub/uc/uc2011/uc2011.pdf

25. Department of Health. 2010-11 reference costs publication. 2011. https://assets.publishing.service.gov.uk/government/uploads/system/uploads/attachment_data/file/216345/dh_122794.pdf

26. Glick H Doshi J, Sonnad S, Polsky D. *Economic evaluation in clinical trials. .* Oxford: Oxford University Press; 2007.

27. Ritchie J. & Spencer L. Qualitative data analysis for applied policy research. In: Bryman A. & Burges R, ed. *Analysing qualitative data.* ; 1994:173–194.

28. Silverman D. *Interpreting qualitative data. methods for analysing talk, text and interaction.* 2nd edn ed. London: Sage Publications; 2001.

29. Robling M, McNamara R, Bennert K, Butler C, Channon S, Cohen D, et al. Training health care practitioners in the 'Talking diabetes' consulting skills intervention: A cluster randomized controlled trial of effects on blood glucose control and quality of life in children with type 1 diabetes (the DEPICTED study). *British Medical Journal*. 2012;26(e2359.):1-17.

For peer review only

30. Price KJ, Wales J, Eiser C, Knowles J, Heller S, Freeman J, Brennan A, McPherson A, Wellington J. Does an intensive self-management structured education course improve outcomes for children and young people with type 1 diabetes? the kids in control OF food (KICk-OFF) cluster-randomised controlled trial protocol. *BMJ Open*. 2013;3(1):e002429.

31. Murphy H, Wadham C, Hassler-Hurst et al, on behalf of Families and Adolescents Communication and Teamwork Study (FACTS) Group. Randomized trial of a diabetes self-management education and family teamwork intervention in adolescents with type 1 diabetes. *Diabetic Medicine*. 2012;29:249-54.

32. Christie D, Thompson R, Sawtell M, Allen E, Cairns J, Smith F, Jamieson E, Hargreaves K, Ingold A, Brooks L, Wiggins M, Oliver S, Jones R,Elbourne D, Santos A, Wong IC, O'Neill S, Strange V, Hindmarsh P, Annan F, Viner R. Structured, intensive education maximising engagement, motivation and long-term change for children and young people with diabetes: A cluster randomised controlled trial with integral process and economic evaluation - the CASCADE study. . *Health Technol Assess*. 2014;18:1-202.

33. Coates V, Chaney D, Bunting B, Shorter GW, Shevlin M, et al. (2013) Evaluation of the Effectiveness of a Structured Diabetes Education Programme (CHOICE) on Clinical Outcomes for Adolescents with Type 1 Diabetes: A Randomised Controlled Trial. J Diabetes Metab 4:280. doi: 10.4172/2155-6156.1000280

34. Charalampopoulos D, Hesketh KR, Amin R, Paes VM, Viner RM, Stephenson T Psycho-educational interventions for children and young people with Type 1 Diabetes in the UK: How effective are they? A systematic review and meta-analysis. PLoS ONE 2017 12(6): e0179685. https://doi.org/10.1371/journal.pone.0179685

35. Lang K, Sassman H, von Schütz W, Kordonouri O, Danne. Prerequisites for age-appropriate education in type 1 diabetes: A model programme for paediatric diabetes education in Germany. *Pediatr Diabetes*. 2007;6:63-74.

36. Mortensen H HP. Comparison of metabolic control in a cross-sectional study of 2,873 children and adolescents with IDDM from 18 countries. the hvidøre study group on childhood diabetes. *Diabetes Care*. 1997;20:714-720.

37. Channon SJ, Huws-Thomas MV, Rollnick S, Hood K, Cannings-John RL, Rogers C, Gregory JW. A Multicenter Randomized Controlled Trial of Motivational Interviewing in Teenagers With Diabetes. Diabetes Care. 2007 Jun;30(6):1390-5)

Competing Interest Statement

Professor John Gregory has received payments from Pfizer, Bayer and Ipsen for lectures, development of educational presentations and travel/accommodation to attend scientific meetings and advisory board meetings. His employer (Cardiff University) has also received funding from Novo Nordisk to support the development of patient-support materials used in the Development and Evaluation of a Psychosocial Intervention for Children and Teenagers Experiencing Diabetes (DEPICTED) research study. The remaining authors declare no competing interests

For peer review only - http://bmjopen.bmj.com/site/about/guidelines.xhtml

Author contributions

JN designed the trial and process evaluation, contributed to intervention development and provided overall study leadership, oversaw data analysis and reanalysis, drafted and critically revised the manuscript.

ITR designed the trial, undertook the trial reanalysis, drafted and critically revised the manuscript.

DA contributed to intervention development, trial and process evaluation design, interpretation of results and critically revised the manuscript.

JWG contributed to intervention development, trial and process evaluation design, interpretation of results and critically revised the manuscript.

RW designed the statistical analysis plan, undertook the analysis and critically revised the manuscript.

YS undertook the statistical analysis and critically revised the manuscript.

RTE designed and conducted the health economic analysis and critically revised manuscript.

STY conducted the health economic analysis and critically revised the manuscript.

DE managed the study on a day to day basis, contributed to data collection and analysis and critically revised the manuscript.

LS managed the study on a day to day basis, contributed to data collection and analysis and critically revised the manuscript.

CC designed and undertook the discourse analysis and critically revised the manuscript.

DR undertook the trial reanalysis and critically revised the manuscript.

Acknowledgements

Emerita Professors Anne Williams and Lesley Lowes. Jan Sharp and Professor Jo Rycroft-Malone. Professor Tim Barrett was independent Chair of the Trial Steering Group. Dr Chris Foy is independent Chair of the Data Monitoring and Ethics Committee (DMEC). Rachel Harris, diabetes nurse specialist was independent member of DMEC. Yvonne Rees-Coleman and Nina Phillips, were independent lay members of the Trial Steering Group. We thank the children, parents and staff who participated in the trial and process evaluation.

Data sharing statement

Trial data is not publicly available but is available upon reasonable request to NWORTH CTU http://nworth-ctu.bangor.ac.uk/

Funding

National Institute for Health Research

Grants: SDO/08/1718/145 and SDO/211/2007.

For peer review only - http://bmjopen.bmj.com/site/about/guidelines.xhtml

Figures

Figure 1. CONSORT diagram from screening to analysis.

Figure 2. CONSORT diagram, showing missing data from screening to analysis

For peer review only

For peer review only - http://bmjopen.bmj.com/site/about/guidelines.xhtml

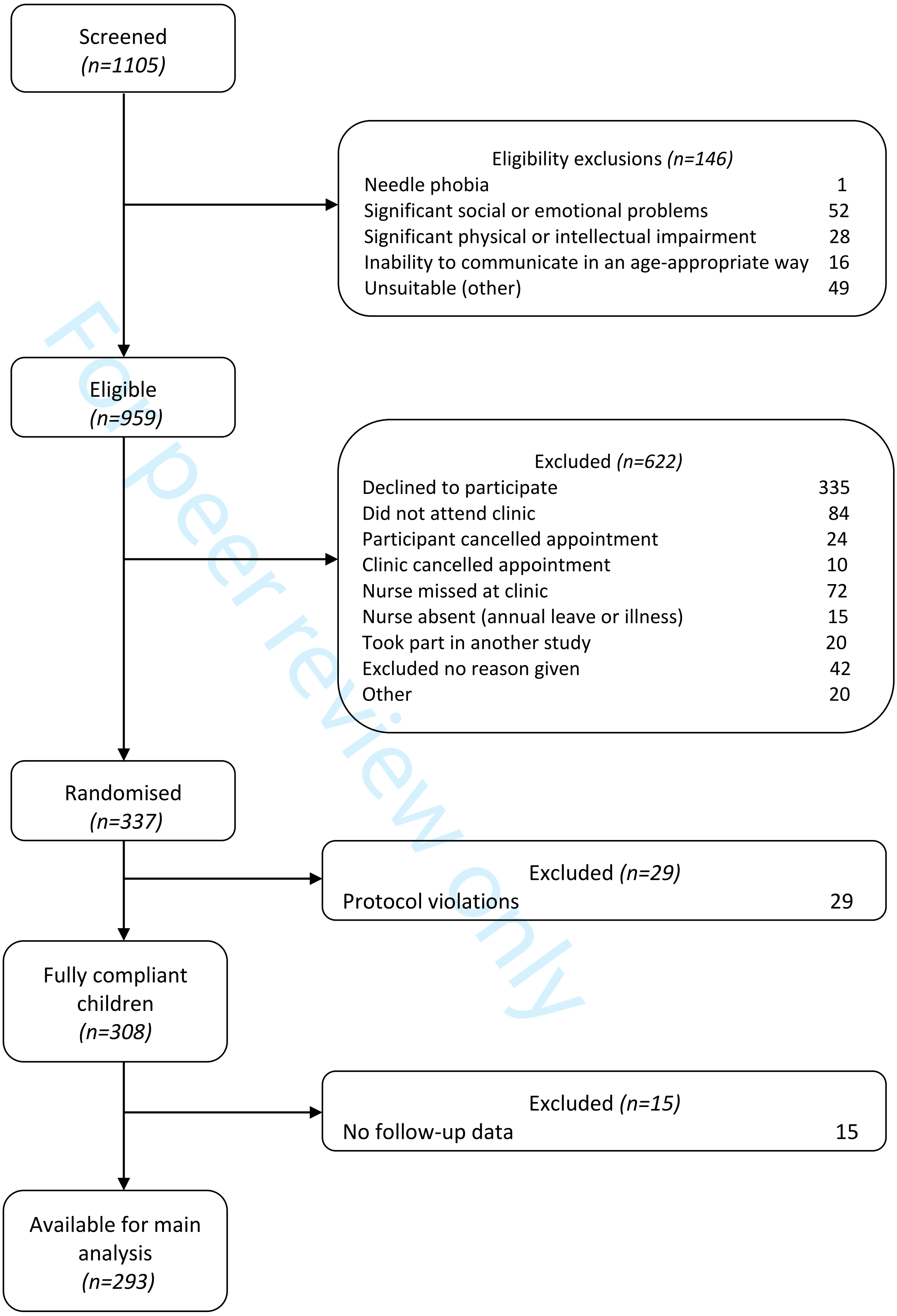

For peer review only

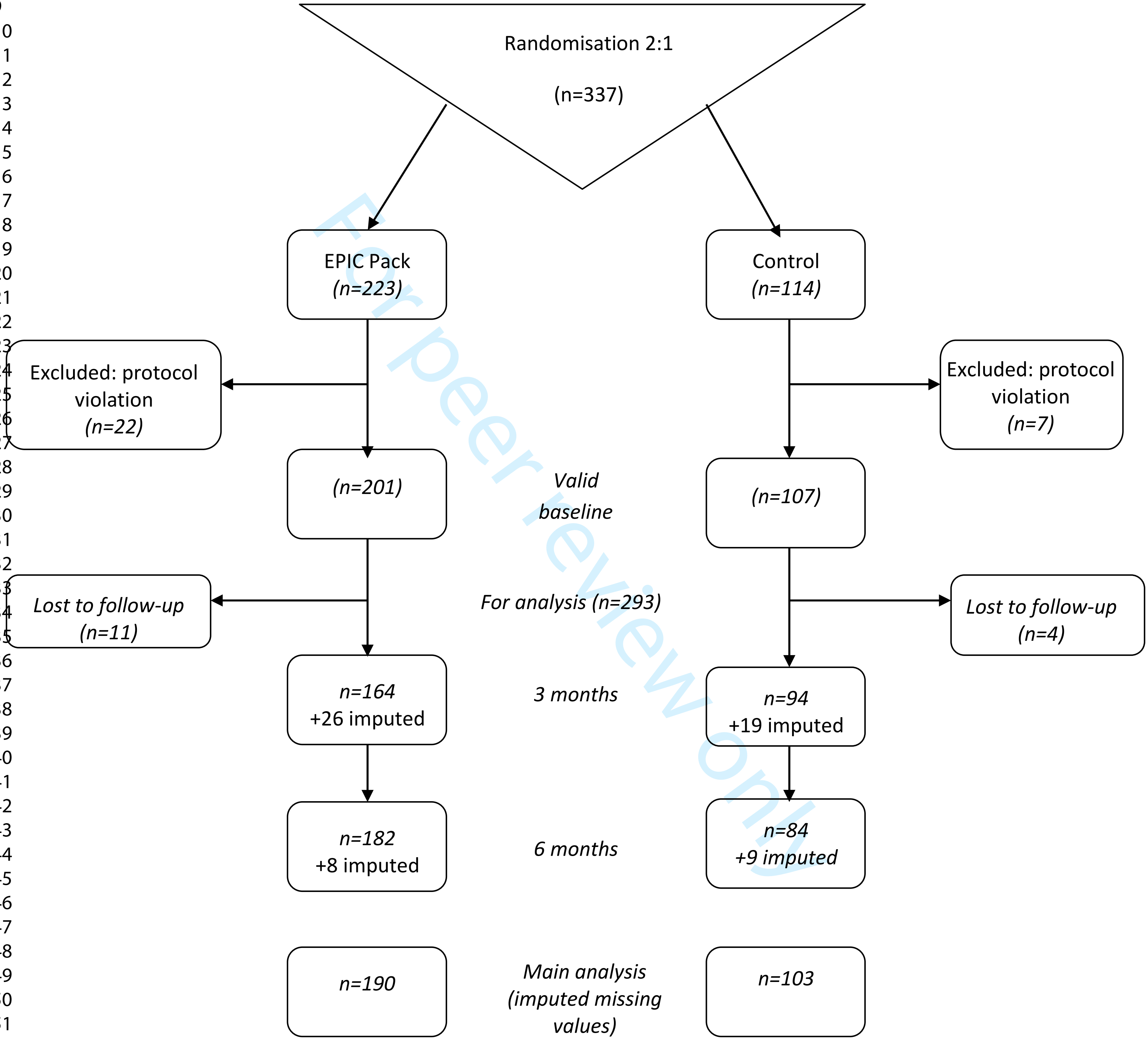

For peer review only

CONSORT 2010 checklist of information to include when reporting a randomised trial*

Section/Topic	Item No	Checklist item	Reported on page No
**Title and abstract**			
	1a	Identification as a randomised trial in the title	No as not just reporting a RCT
	1b	Structured summary of trial design, methods, results, and conclusions (for specific guidance see CONSORT for abstracts)	2-3
**Introduction**			
Background and objectives	2a	Scientific background and explanation of rationale	5-6
	2b	Specific objectives or hypotheses	5
**Methods**			
Trial design	3a	Description of trial design (such as parallel, factorial) including allocation ratio	6-10
	3b	Important changes to methods after trial commencement (such as eligibility criteria), with reasons	
Participants	4a	Eligibility criteria for participants	7
	4b	Settings and locations where the data were collected	7
Interventions	5	The interventions for each group with sufficient details to allow replication, including how and when they were actually administered	6-7
Outcomes	6a	Completely defined pre-specified primary and secondary outcome measures, including how and when they were assessed	8
	6b	Any changes to trial outcomes after the trial commenced, with reasons	
Sample size	7a	How sample size was determined	9
	7b	When applicable, explanation of any interim analyses and stopping guidelines	
Randomisation:			
Sequence generation	8a	Method used to generate the random allocation sequence	7
	8b	Type of randomisation; details of any restriction (such as blocking and block size)	7
Allocation concealment mechanism	9	Mechanism used to implement the random allocation sequence (such as sequentially numbered containers), describing any steps taken to conceal the sequence until interventions were assigned	8
Implementation	10	Who generated the random allocation sequence, who enrolled participants, and who assigned participants to	7

For peer review only

		interventions	
Blinding	11a	If done, who was blinded after assignment to interventions (for example, participants, care providers, those assessing outcomes) and how	8
	11b	If relevant, description of the similarity of interventions	
Statistical methods	12a	Statistical methods used to compare groups for primary and secondary outcomes	9
	12b	Methods for additional analyses, such as subgroup analyses and adjusted analyses	9
**Results**			
Participant flow (a diagram is strongly recommended)	13a	For each group, the numbers of participants who were randomly assigned, received intended treatment, and were analysed for the primary outcome	11
	13b	For each group, losses and exclusions after randomisation, together with reasons	11 and figures
Recruitment	14a	Dates defining the periods of recruitment and follow-up	2
	14b	Why the trial ended or was stopped	
Baseline data	15	A table showing baseline demographic and clinical characteristics for each group	12-13
Numbers analysed	16	For each group, number of participants (denominator) included in each analysis and whether the analysis was by original assigned groups	Figure 1
Outcomes and estimation	17a	For each primary and secondary outcome, results for each group, and the estimated effect size and its precision (such as 95% confidence interval)	15-18
	17b	For binary outcomes, presentation of both absolute and relative effect sizes is recommended	
Ancillary analyses	18	Results of any other analyses performed, including subgroup analyses and adjusted analyses, distinguishing pre-specified from exploratory	18
Harms	19	All important harms or unintended effects in each group (for specific guidance see CONSORT for harms)	14
**Discussion**			
Limitations	20	Trial limitations, addressing sources of potential bias, imprecision, and, if relevant, multiplicity of analyses	25
Generalisability	21	Generalisability (external validity, applicability) of the trial findings	25-6
Interpretation	22	Interpretation consistent with results, balancing benefits and harms, and considering other relevant evidence	25-6
**Other information**			
Registration	23	Registration number and name of trial registry	2
Protocol	24	Where the full trial protocol can be accessed, if available	6
Funding	25	Sources of funding and other support (such as supply of drugs), role of funders	30

For peer review only - http://bmjopen.bmj.com/site/about/guidelines.xhtml

*We strongly recommend reading this statement in conjunction with the CONSORT 2010 Explanation and Elaboration for important clarifications on all the items. If relevant, we also recommend reading CONSORT extensions for cluster randomised trials, non-inferiority and equivalence trials, non-pharmacological treatments, herbal interventions, and pragmatic trials. Additional extensions are forthcoming: for those and for up to date references relevant to this checklist, see www.consort-statement.org.

For peer review only
