# Supplementary material for: Standardised self-management kits for children with type 1 diabetes: pragmatic randomised trial of effectiveness and cost-effectiveness
Source: BMJ Open. 2020 Mar 12;10(3):e032163. doi: 10.1136/bmjopen-2019-032163 (PMC7069268; doi:10.1136/bmjopen-2019-032163)
Supplement: Reviewer comments [file bmjopen-2019-032163.reviewer_comments.pdf]

PEER REVIEW HISTORY

BMJ Open publishes all reviews undertaken for accepted manuscripts. Reviewers are asked to complete a checklist review form (**http://bmjopen.bmj.com/site/about/resources/checklist.pdf**) and are provided with free text boxes to elaborate on their assessment. These free text comments are reproduced below.

This paper was submitted to a another journal from BMJ but declined for publication following peer review. The authors addressed the reviewers' comments and submitted the revised paper to BMJ Open. The paper was subsequently accepted for publication at BMJ Open.

ARTICLE DETAILS

**TITLE (PROVISIONAL)**	Standardised self-management kits for children with type 1 diabetes: pragmatic randomised trial of effectiveness and cost-effectiveness
**AUTHORS**	Noyes, Jane; Allen, Davina; Carter, Cynthia; Edwards, Deborah; Edwards, Rhiannon; Russell, Daphne; Russell, Ian; Spencer, Llinos; Sylvestre, Yvonne; Whitaker, Rhiannon; Yeo, S. T.; Gregory, John
